# Raddeanin A Enhances Mitochondrial DNA‐cGAS/STING Axis‐Mediated Antitumor Immunity by Targeting Transactive Responsive DNA‐Binding Protein 43

**DOI:** 10.1002/advs.202206737

**Published:** 2023-03-06

**Authors:** Mingxiao Yin, Jingwen Dong, Cuicui Sun, Xiaojia Liu, Zhirui Liu, Lu Liu, Zean Kuang, Na Zhang, Dian Xiao, Xinbo Zhou, Hongbin Deng

**Affiliations:** ^1^ Institute of Medicinal Biotechnology Chinese Academy of Medical Sciences & Peking Union Medical College Beijing 100050 P. R. China; ^2^ Beijing Institute of Clinical Pharmacy Beijing Friendship Hospital Capital Medical University Beijing 100050 P. R. China; ^3^ National Engineering Research Center for the Emergency Drug Beijing Institute of Pharmacology and Toxicology Beijing 100850 P. R. China; ^4^ Qingdao Women and Children's Hospital Qingdao University Qingdao 266034 P. R. China

**Keywords:** dendritic cells, immune checkpoint therapies, immunogenic cell death, STING, transactive responsive DNA‐binding protein 43

## Abstract

Immune checkpoint therapies (ICT) have achieved unprecedented efficacy in multiple cancer treatments, but are still limited by low clinical response rates. Identification of immunogenic cell death (ICD)‐inducing drugs that can induce tumor cell immunogenicity and reprogram the tumor microenvironment is an attractive approach to enhance antitumor immunity. In the present study, Raddeanin A (RA), an oleanane class triterpenoid saponin isolated from *Anemone raddeana Regel*, is uncovered as a potent ICD inducer through an ICD reporter assay combined with a T cell activation assay. RA significantly increases high‐mobility group box 1 release in tumor cells and promotes dendritic cell (DC) maturation and CD8^+^ T cell activation for tumor control. Mechanistically, RA directly binds to transactive responsive DNA‐binding protein 43 (TDP‐43) and induces TDP‐43 localization to mitochondria and mtDNA leakage, leading to cyclic GMP‐AMP synthase/stimulator of interferon gene‐dependent upregulation of nuclear factor *κ*B and type I interferon signaling, thereby potentiating the DC‐mediated antigen cross‐presentation and T cell activation. Moreover, combining RA with anti‐programmed death 1 antibody effectively enhances the efficacy of ICT in animals. These findings highlight the importance of TDP‐43 in ICD drug‐induced antitumor immunity and reveal a potential chemo‐immunotherapeutic role of RA in enhancing the efficacy of cancer immunotherapy.

## Graphical abstract

Raddeanin A (RA) is uncovered as a potent immunogenic cell death inducer that reprograms the tumor microenvironment. RA stimulates cyclic GMP‐AMP synthase/stimulator of interferon gene‐dependent type I interferon signaling in tumors by directly binding to transactive responsive DNA‐binding protein 43, thereby triggering dendritic cell‐mediated antigen cross‐presentation and T cell activation.

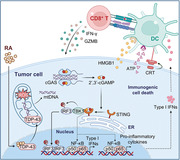

## 1 Introduction

Cancer cells often bypass immune surveillance through suppressing the function of effector T cell. T cell‐based immune checkpoint therapies (ICT), such as cytotoxic T lymphocyte‐associated antigen 4 (CTLA‐4), programmed death 1 (PD‐1)/programmed death ligand 1 (PD‐L1) blockade, have demonstrated impressive clinical efficacy in reviving the antitumor function of exhausted T cells and providing long‐lasting protection across various cancer types.^[^ ^1^ ^]^ Despite these successes, ICT is still limited by low clinical response rates, lack of durable remission and immune‐related adverse events.^[^ ^2^ ^]^ Multiple lines of evidences have shown that systemic anticancer immunity can be reinstated to increase the antitumor efficacy of ICT via induction of immunogenic cell death (ICD), a non‐conventional type of apoptosis that is associated with the activation of an adaptive immune response.^[^ ^3^, ^4^ ^]^ Clinical evidences also support the importance of ICD for long‐term therapeutic effects as patients bearing tumors that lack features of ICD‐related immunogenic factors have a poor prognosis.^[^ ^4^ ^]^ ICD‐inducing drugs may enhance tumor antigen exposure, boost the release of immune‐stimulating tumor cell content, and elicit immune cell infiltration.^[^ ^4^, ^5^ ^]^ Therefore, identification of drugs that can both enhance tumor immunogenicity and potentiate efficacy of ICT is an unmet clinical challenge.

Recent studies have shown that radiation therapy and certain classes of chemotherapies elicit antitumor immunity by inducing tumor cell ICD.^[^ ^5^, ^6^, ^7^ ^]^ These treatments induce tumor cell dying and upregulate the expression or release of damage‐associated molecular patterns (DAMPs) such as adenosine triphosphate (ATP), calreticulin (CRT), and high‐mobility group box1 protein (HMGB1).^[^ ^8^ ^]^ ATP functions as chemoattractant signal for dendritic cells (DCs) precursors, while CRT acts as an “eat me” signal to facilitate phagocytosis of DCs and trigger antigen‐specific T cell responses.^[^ ^9^ ^]^ Meanwhile, liberated HMGB1 activates toll‐like receptor‐4 to stimulate DCs maturation.^[^ ^10^ ^]^ Additionally, tumor cell ICD‐induced inflammatory cytokines and chemokines production are associated with upregulation of nuclear factor *κ*B (NF‐*κ*B) and type I interferon (IFN) signaling,^[^ ^11^, ^12^ ^]^ which in turn promote DCs and T cell activation and facilitate tumor eradication. Thus, the ICD‐inducing chemotherapeutic drugs may potentiate the therapeutic efficacy of immunotherapy by killing cancer cells as well as augmentation of host immune system.^[^ ^3^, ^5^ ^]^

The human transactive responsive (TAR) DNA‐binding protein 43 (TDP‐43) is a nuclear DNA/RNA binding protein which involved in a number of diseases such as amyotrophic lateral sclerosis (ALS), Alzheimer's disease (AD), and cancers.^[^ ^13^, ^14^ ^]^ TDP‐43 is primarily located in the nucleus of mammalian cells and is known to shuttle between the cell nucleus and cytoplasm.^[^ ^15^ ^]^ Aside from two RNA binding domains and a low‐complexity glycine‐rich region, TDP‐43 also encodes a nuclear localization sequence and nuclear export sequence, which mediate its shuttling between the nucleus and cytosol.^[^ ^16^ ^]^ Recent studies show that the missense mutations (e.g., A315T, Q331K) in the C‐terminal glycine‐rich domain of TDP‐43 induces TDP‐43 to invade mitochondria and releases mitochondrial DNA (mtDNA) via the permeability transition pore thus triggers cyclic GMP‐AMP synthase/stimulator of interferon gene (cGAS/STING) activation.^[^ ^17^ ^]^ Therefore, mislocalized TDP‐43 affects homeostasis of the cell and could have the consequences for triggering immune response pathways.

Inspired by the bioactive substances of natural medicines in cancer treatment, we used an ICD reporter assay and a T cell activation assay^[^ ^18^ ^]^ to screen for ICD‐inducing compounds from a natural product library, and identified Raddeanin A (RA) as a novel anticancer agent that specifically induced tumor cell ICD. RA, an oleanane class triterpenoid saponin isolated from *Anemone raddeana Regel*,^[^ ^19^ ^]^ was not previously demonstrated to affect tumor cell ICD. Here, we provided evidences that RA potentially enhanced tumor immunogenicity and promoted DCs and T cells activation. RA inhibited tumor growth in vivo in a manner dependent on DCs and CD8^+^ T cells activation. More importantly, RA stimulated cGAS/STING‐dependent NF‐*κ*B and type I IFN signaling by binding to TDP‐43, thereby triggered the DCs‐mediated antigen cross‐presentation and T cells activation, which together boosted antitumor immunity. Our study collectively provides experimental evidences illustrating a potential chemo‐immunotherapeutic role of RA to enhance the efficacy of cancer immunotherapy.

## 2 Results

### 2.1 Identification of Raddeanin A as a Potent Immunogenic Cell Death‐Inducing Chemical

**Figure 1:**
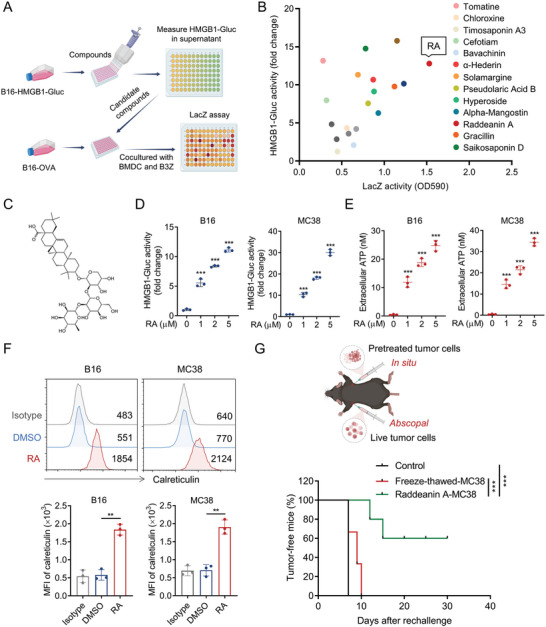
RA is a potent ICD inducer. A) Outline of chemicals screening protocol. Natural compounds were screened by HMGB1‐Gluc reporter assay, the hit compounds were then evaluated by LacZ T cell reporter assay. B) Scatter diagram presentation of the influence of hit compounds on HMGB1 release and LacZ T cell activation. C) Chemical structure of RA. B16‐HMGB1‐Gluc and MC38‐HMGB1‐Gluc cells were treated with indicated doses of RA for 20 h, D) HMGB1‐Gluc luciferase activity and E) extracellular ATP levels were measured. F) B16 and MC38 cells were treated with DMSO or RA (5 µM) for 20 h, and surface expressions of CRT were detected by fluorescence activated cell sorting (FACS). G) MC38 cells were pretreated with DMSO, RA, or freeze‐thawed, followed by subcutaneous inoculation into C57BL6 mice as a vaccine (*n* = 8). After 7 days, mice were rechallenged with live MC38 cells. The percentages of tumor‐free mice 30 days after rechallenge are shown on the bottom. Data in (D)–(F) are shown as mean ± SD of 3 independent experiments. ^**^ *p* < 0.01, ^***^ *p* < 0.001, D,E) one‐way ANOVA test, F) unpaired Student's *t*‐test, G) log‐rank (Mantel‐Cox) test.

To identify novel compounds that could induce tumor cell death and boost T cell response, we performed chemical screenings based on an ICD reporter assay and a T cell activation assay.^[^ ^18^ ^]^ The ability for a natural bioactive chemical library (Selleck L1400) to elicit the release of HMGB1, one of the hallmarks of ICD, was evaluated by a HMGB1‐Gluc reporter assay.^[^ ^18^ ^]^ Next, the OVA‐expressing B16 mouse melanoma cells (B16‐OVA) cocultured with marrow‐derived DCs (BMDCs) and B3Z (a CD8^+^ T cell hybridoma specifically recognize OVA epitope) cells were used to screen for hit chemicals that could activate CD8^+^ T cells (**Figure** **1**A).^[^ ^18^ ^]^ Screening data were translated into a scatter diagram representing the relative change of HMGB1‐Gluc and LacZ activities responses to chemicals (Figure 1B). Interestingly, we found RA, an oleanane class triterpenoid saponin isolated from *Anemone raddeana Regel*, induced the highest level of HMGB1‐Gluc and LacZ activities (Figure 1B,C).

We next attempted to further validate the ability of RA to stimulate ICD of tumor cells. RA treatment induced a significant increase of HMGB1‐Gluc activity (Figure 1D) and ATP release (Figure 1E) in a dose‐dependent manner in both B16 and MC38 cells. Moreover, RA treatment dramatically upregulated cell surface expression of CRT, another known ICD marker that serve as “eat‐me” signal for tumor immune recognition (Figure 1F), further confirming the ICD‐inducing effect of RA. In line with previous report,^[^ ^20^ ^]^ RA induced markedly apoptosis in MC38 and B16 cells (Figure S1A, Supporting Information), while blockade of apoptosis by a pan‐caspase inhibitor (Z‐VAD‐FMK), but not necroptosis inhibitor (necrostatin1) or lysosomal cell death inhibitor CA‐074 Me, inhibited RA‐induced HMGB1, ATP release and CRT surface expressions in tumor cells (Figure S1B–G, Supporting Information), indicating RA triggered ICD effects of tumor cells mainly through induction of apoptosis. To further validate the immunogenicity‐inducing effect of RA in vivo, we inoculated C57BL/6 mice with RA‐pretreated MC38 dead cells or freeze‐thawed cells on the right flank (in situ). The mice were rechallenged with live untreated MC38 cells on the left flank (abscopal Figure 1G) 7 days later. We found the mice treated with DMSO or vaccinated with freeze‐thawed MC38 cells all developed tumors, while mice immunized with RA‐treated dead MC38 cells displayed 60% tumor‐free survival in the 30 days abscopal challenge (Figure 1G). Collectively, these results confirmed RA as a potent ICD inducer.

### 2.2 Raddeanin A‐Treated Tumor Cells Promote Dendritic Cells‐Mediated T Cells Activation

**Figure 2:**
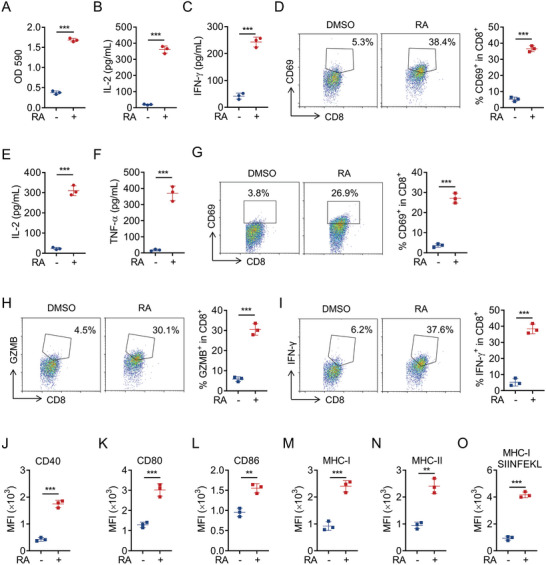
RA‐treated tumor cells promote DCs‐mediated T cells activation. B16‐OVA cells were treated with DMSO or RA (5 µM) for 20 h, followed by coculturing with BMDCs and B3Z cells for an additional 24 h, then the activation of B3Z cells were measured by A) LacZ activity, B) secretion of IL‐2, and C) IFN‐*γ*, and D) surface expression of CD69. B16‐OVA cells were treated with DMSO or RA (5 µM) for 20 h, followed by coculturing with BMDCs and OT‐I cells for an additional 24 h, then OT‐I activation was examined by E) IL‐2 and F) TNF‐*α* production, G) surface expression of CD69, H) effector molecules GZMB, and I) IFN‐*γ* production. J–O) B16‐OVA cells were treated with DMSO or RA (5 µM) for 20 h, then cocultured with BMDCs for an additional 24 h, after which surface expression of CD40, CD80, CD86, MHC‐I, MHC‐II, and MHC‐I‐SIINFEKL on CD11c^+^ BMDCs was determined by FACS. Data are shown as mean ± SEM of 3 independent experiments. A–O) ^**^ *p* < 0.01, ^***^ *p* < 0.001, unpaired Student's *t*‐test.

We next determined the ability of RA‐treated tumor cells to activate T cells and DCs. B16‐OVA cells were treated with DMSO or RA for 20 h, followed by coculturing with BMDCs and B3Z T cells for 24 h. We found RA treatment significantly increased LacZ activity in B3Z T cells (**Figure** **2**A). In addition, the supernatant levels of T cell‐derived cytokines interleukin‐2 (IL‐2) and interferon‐γ (IFN‐*γ)* were remarkably upregulated in B3Z T cells cocultured with RA‐pretreated tumor cells (Figure 2B,C), concurrently accompanied with increased expression level of T cell activation marker CD69 (Figure 2D). Consistent with results, we observed similar results when primary OT‐I T cells were used instead of B3Z T cells. The supernatant levels of IL‐2 and tumor necrosis factor‐α (TNF‐*α)* were significantly increased in OT‐I T cells cocultured with RA‐pretreated tumor cells (Figure 2E,F). Meanwhile, the expression level of CD69 (Figure 2G) and effector molecules Granzyme B (GZMB) and IFN‐*γ* (Figure 2H,I) also dramatically increased after coculture. As DCs play a key role in cross‐presentation of tumor antigen to T cells,^[^ ^21^ ^]^ and RA‐treated‐B16‐OVA cells did not directly activate T cells without BMDCs (Figure S2A,B, Supporting Information), we next examined the activation status of DCs cocultured with RA‐treated tumor cells. RA‐treated B16‐OVA tumor cells markedly increased the surface expression of activation markers, including CD40, CD80, CD86, MHC‐I, and MHC‐II on BMDCs (Figure 2 J–N). Similar results were observed in MC38‐OVA cells (Figure S2C–G, Supporting Information). Moreover, the surface expression level of MHC‐I‐bound SIINFEKL complex also significantly increased by RA treatment (Figure 2O; Figure S2H, Supporting Information). The above data suggest that RA‐treated tumor cells promote DCs maturation and subsequent CD8^+^ T cells activation.

### 2.3 Raddeanin A Inhibited Tumor Growth via Activating Antitumor Immunity

**Figure 3:**
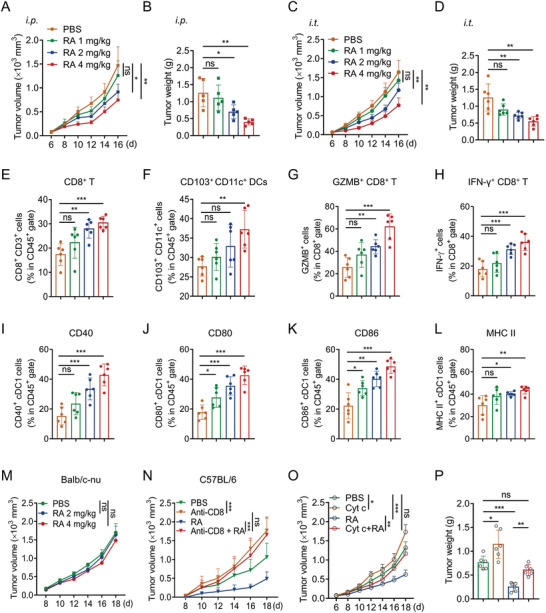
RA mediates a DCs and T cell dependent antitumor effect. C57BL/6 mice with subcutaneous MC38 tumor were i.p. or i.t. injected with PBS or RA (1, 2, and 4 mg kg^−1^), and the A,C) tumor volume and B,D) tumor weight were monitored for 16 days. FACS analyzing the populations of tumor‐infiltrating E) CD8^+^ T cells, F) CD103^+^CD11c^+^ DCs, and G) effector molecules GZMB, and H) IFN‐*γ* in CD8^+^ T cells from each group. I–L) Surface expression levels of CD40, CD80, CD86, and MHC‐II on CD103^+^ CD11c^+^ cells from each group were determined by FACS. *n* = 6 mice per group. M) Balb/c nude mice bearing MC38 tumor were received with PBS or RA (2, 4 mg kg^−1^, i.t.) four times at the indicated time points, and the tumor growth was measured. *n* = 7 per group. N) C57BL6 mice bearing MC38 tumor were received with PBS, anti‐CD8 neutralizing antibody, and/or RA (4 mg kg^−1^, i.t.), and the tumor growth was measured. *n* = 7 per group. O,P) C57BL6 mice bearing MC38 tumor were treated with PBS, Cyt c that deplete DCs, and/or RA (4 mg kg^−1^, i.t.). O) Tumor volume and P) tumor weight are shown as mean ± SD. *n* = 7 per group. A,CM–O) Data are presented as mean ± SD, ^*^ *p* < 0.05, ^**^ *p* < 0.01, ^***^ *p* < 0.001, ns, not significant, two‐way ANOVA test, B,D,E–L,P) one‐way ANOVA test.

To examine the impact of RA on the immune control of tumor growth in vivo, we subcutaneously inoculated MC38 melanoma cells into C57BL/6 mice and monitored the tumor growth (Figure S3A,B, Supporting Information). We found intraperitoneally (i.p.) or intratumorally (i.t.) injection of RA (1, 2, and 4 mg kg^−1^) caused considerable tumor size and tumor weight inhibition on MC38 tumor‐bearing mice (**Figure** **3**A–D; Figure S3A,B, Supporting Information). Similarly, RA effectively suppressed tumor growth in B16 melanoma‐bearing mice (Figure S3C,D, Supporting Information). Meanwhile, i.t. treatment with RA did not remarkably alter the mice body weight (Figure S3E, Supporting Information), blood biochemical alanine aminotransferase (ALT) and aspartate aminotransferase (AST) levels (Figure S3F, Supporting Information) or organ indexes including heart, liver, spleen, and lung (Figure S3G, Supporting Information). Additionally, tumor‐infiltrating lymphocyte (TILs) profile analysis revealed that RA markedly elevated the population of tumor‐infiltrating CD8^+^ T cells, and CD103^+^ CD11^+^ DCs, generally considered type 1 conventional dendtritic cells (cDC1) (Figure 3E,F; Figure S4A,B, Supporting Information). Meanwhile, the levels of CD8^+^ T cell effector molecules GZMB and IFN‐*γ* within tumor microenvironment (TME) also dramatically increased in RA‐treated group (Figure 3G,H; Figure S4C,D, Supporting Information), implying that RA enhances the activation of effective T cells in vivo. Consistent with the in vitro results, MC38‐tumor‐infiltrating cDC1 also showed upregulated CD40, CD80, CD86, and MHC‐II expression after RA treatment (Figure 3I–L; Figure S4E–H, Supporting Information).

Interestingly, in BALB/c nude mice with defective T cell function, RA failed to suppress the tumor growth of MC38 (Figure 3M). In support of this notion, anti‐CD8 depletion antibody attenuated RA‐induced MC38 tumor inhibition in C57BL/6 mice (Figure 3N), confirming a critical role of CD8 T cell in RA‐mediated antitumor immunity. To further determine the role of DCs in RA‐induced antitumor effect, we depleted DCs by cytochrome c (Cyt c) in vivo.^[^ ^22^ ^]^ Intravenous injections of Cyt c successfully eliminated splenic DCs and tumor associated DCs in mice (Figure S4I,J, Supporting Information). Moreover, DCs‐depletion by Cyt c abolished the inhibition of MC38 tumor growth by RA (Figure 3O,P), suggesting that antigen presentation was essential for RA‐mediated antitumor effect. Taking together, these results showed that RA could inhibit tumor growth through a DCs and CD8^+^ T cell‐dependent manner.

### 2.4 Raddeanin A‐Induced Tumor Cell Immunogenicity is Dependent on cGAS‐STING

**Figure 4:**
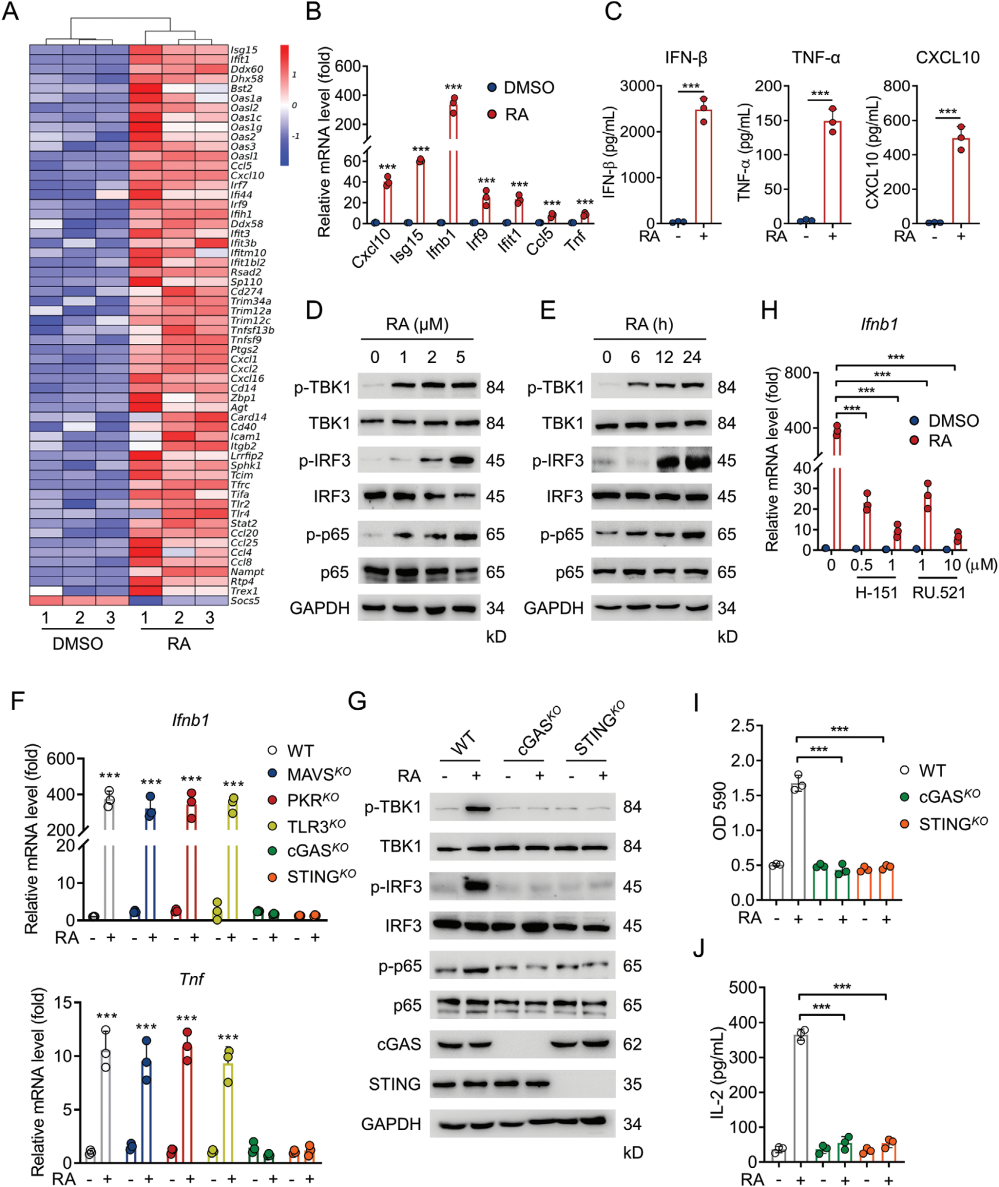
RA triggers cGAS/STING‐dependent NF‐*κ*B and type I IFN signaling activation. A) RNA‐seq analysis of genes differentially expressed in MC38 cells treated with DMSO or RA (5 µM). B) qPCR analyzing NF‐*κ*B and type I IFN pathways associated genes in MC38 cells treated with DMSO or RA (5 µM) for 24 h. C) Enzyme‐linked immunosorbent assay (ELISA) determining the IFN‐*β*, TNF‐*α*, and CXCL10 levels in the supernatant of MC38 cells treated with DMSO or RA (5 µM) for 24 h. Immunoblotting (IB) analysis of the phosphorylation levels of TBK1, IRF3, and p65 in MC38 cells treated D) with indicated concentrations of RA for 24 h, or E) with RA (5 µM) for different time. F) Innate immune sensors in MC38 cells were knockout by sgRNA, the expressions of Ifnb1 and Tnf were examined by qPCR after treatment with DMSO or RA (5 µM) for 24 h. G) IB analysis of the phosphorylation levels of TBK1, IRF3, and p65 in WT, cGAS^KO^ or STING^KO^ MC38 cells treated with DMSO or RA (5 µM) for 24 h. H) qPCR analysis of the expression of Ifnb1 in DMSO or RA‐treated MC38 cells in the presence of cGAS inhibitor RU.521 and STING inhibitor H‐151. WT, cGAS^KO^, or STING^KO^ B16 cells were treated with DMSO or RA (5 µM) for 24 h, followed by coculturing with BMDCs and B3Z cells for an additional 24 h, I) LacZ activity and J) IL‐2 production were determined. Data are shown as mean ± SEM of 3 independent experiments. ^***^ *p* < 0.001, B,C) unpaired Student's *t*‐test, F,H,I,J) two‐way ANOVA test.

To interrogate how RA induced tumor cell ICD, we profiled RNA sequencing (RNA‐seq) analysis to identify different gene expression patterns in DMSO or RA treated‐MC38 cells. Interestingly, NF‐*κ*B and type I IFN signaling‐associated genes were found to be significantly enriched by RA treatment (**Figure** **4**A). Meanwhile, quantitative real‐time PCR (qPCR) confirmed the upregulation of NF‐*κ*B and type I IFN pathway genes such as Cxcl10, Isg15, Ifnb1, Ccl5, Tnf, Irf9, Ifit1 in MC38 (Figure 4B) and B16 cells (Figure S5A, Supporting Information). Reporter gene assay also suggested that RA triggered NF‐*κ*B and type I IFN pathway activation in MC38 cells (Figure S5B, Supporting Information). Moreover, RA treatment remarkably increased the protein levels of NF‐*κ*B and type I IFN pathway downstream cytokines such as IFN‐*β*, TNF‐*α*, and CXCL10 (Figure 4C). At the molecular level, RA treatment increased the phosphorylation levels of downstream signaling molecules TBK1, IRF3, and p65 in a dose and time‐dependent manner in MC38 (Figure 4D,E) and B16 cells (Figure S5C,D, Supporting Information).

To identify the innate immune sensor regulating RA‐mediated NF‐*κ*B and type I IFN activation, we genetically deleted a panel of candidates including RIG‐I, MDA‐5, cGAS, STING, PKR, and TLR3 that are known to regulate NF‐*k*B and type I IFN production.^[^ ^23^, ^24^, ^25^ ^]^ Interestingly, CRISPR‐mediated deletion of the cytoplasmic RNA sensors including RIG‐I and MDA‐5 (via deletion of the conserved signaling adaptor MAVS),^[^ ^24^ ^]^ PKR,^[^ ^26^ ^]^ and TLR3,^[^ ^23^ ^]^ did not reduce RA‐induced type I IFN or NF‐kB activation (Figure 4F; Figure S5E, Supporting Information) and the phosphorylation of TBK1, IRF3, and p65 (Figure S5F,G, Supporting Information). Instead, absence of the cytoplasmic DNA sensors cGAS and STING,^[^ ^25^ ^]^ led to significant attenuation of NF‐*k*B and type I IFN pathways, as evidenced by Ifnb1 and Tnf gene expression (Figure 4F; Figure S5E, Supporting Information) and activation of signaling molecules including phosphorylation of TBK1, IRF3, and p65 in MC38 and B16 cells (Figure 4G; Figure S5H, Supporting Information). In addition, pharmacological blockade of cGAS /STING pathway by specific inhibitors of cGAS (RU.521)^[^ ^27^ ^]^ and STING (H‐151)^[^ ^28^ ^]^ prevented the expression of Ifnb1 and Tnf in RA‐treated MC38 cells (Figure 4H). Moreover, RA‐induced HMGB1‐Gluc activities were abolished in cGAS^KO^ or STING^KO^ B16 cells (Figure S5I, Supporting Information), and RA treatment in cGAS^KO^ or STING^KO^ B16‐OVA cells could not further activate LacZ reporter activity (Figure 4I) and IL‐2 release (Figure 4J) in B3Z cells in the presence of DCs. Furthermore, RA failed to suppress the tumor growth of mice bearing cGAS^KO^ or STING^KO^ MC38 cells (Figure S5J, Supporting Information). Collectively, these results implicate cGAS/STING as an important immune sensor regulating RA‐mediated tumor cell immunogenicity.

### 2.5 Raddeanin A Directly Binds to Transactive Responsive DNA‐Binding Protein 43

**Figure 5:**
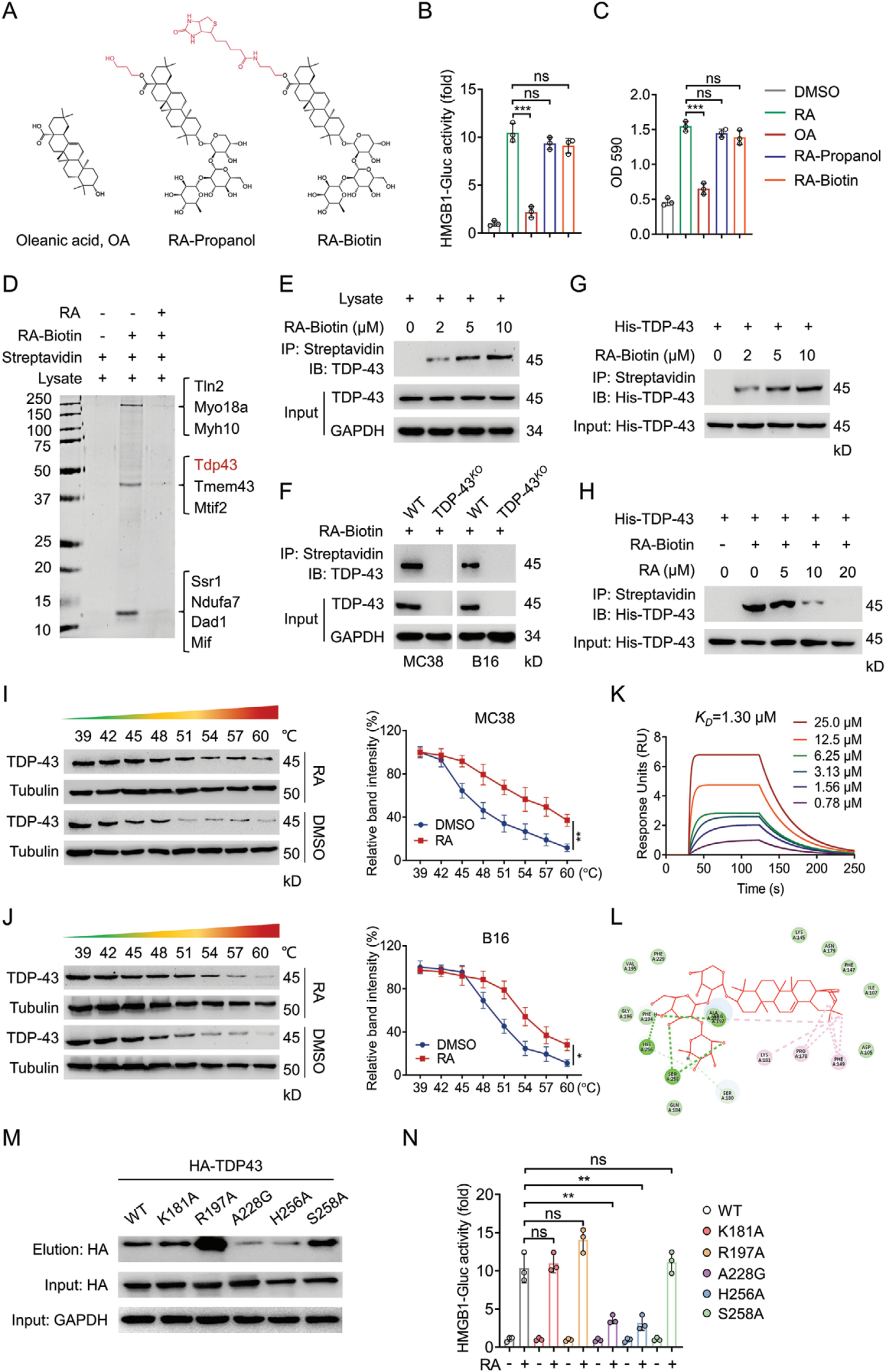
RA directly binds to TDP‐43. A) The chemical structures of analogues of RA and RA‐biotin. B) B16‐HMGB1‐Gluc cells were treated with RA or its analogues for 20 h, HMGB1‐Gluc release was measured. C) B16‐OVA cells were treated with RA or its analogues for 24 h, followed by coculturing with BMDCs and B3Z cells for an additional 24 h, LacZ activity was measured. D) MC38 cell lysates were incubated with RA‐biotin with or without RA at 4 °C overnight, the lysates were subjected to streptavidin‐agarose pull‐down assays, and the precipitates were resolved by SDS‐PAGE and silver staining. The indicated bands were excised and analyzed by MS. IB analysis of the streptavidin‐agarose precipitated TDP‐43 from E) MC38 cells or F) TDP‐43^KO^ MC38 or B16 cells incubated with RA‐Biotin at 4 °C overnight. G) The recombinant His‐TDP‐43 proteins were incubated with indicated RA‐biotin in the absence or presence of a H) two‐fold excess of unlabeled RA at 4 °C overnight, the mixtures were precipitated by streptavidin‐agarose and immunoblotted with His antibody. I,J) CETSA analysis of TDP‐43 target engagement by RA in MC38 cells and B16 cells. Quantifications of the thermal stability of TDP‐43 were indicated on the right. K) SPR analysis of the RA and TDP‐43 binding. The activated SR7000 sensor chip was used to immobilize the recombinant mouse TDP‐43 protein and flowed across RA. L) Molecular docking model revealing RA binds to the RRM domain of TDP‐43. M) 293T cells were transfected with TDP‐43‐WT and TDP‐43‐mutant plasmids for 48 h, cell lysates were incubated with RA‐biotin at 25 °C for 2 h, followed by pull‐down with streptavidine‐agarose; the precipitates were then immunoblotted by HA antibody. N) TDP‐43 WT, TDP43‐mutant plasmids were introduced into TDP‐43^KO^ B16 cells, the HMGB1‐Gluc activity were determined after treatment with DMSO or RA (5 µM) for 24 h. Data are presented as mean ± SD, ^*^ *p* < 0.05, ^**^ *p* < 0.01, ^***^ *p* < 0.001, ns, not significant, B,C) one‐way ANOVA test, I,J,N) two‐way ANOVA test.

To explore the functional target(s) of RA associated with cGAS/STING pathway, we first prepared RA analogues to analyze the roles of carboxyl group and sugar moieties in the action of RA (**Figure** **5**A). HMGB1‐Gluc reporter assay revealed that the carboxyl group (—COOH) analogue RA‐propanol fully retained the biological activity of RA, while the sugar moieties‐depleted analogue oleanic acid (OA) totally abolished the biological activity of RA (Figure 5B; Figure S6A, Supporting Information). This finding suggested that a biotin tag could be attached to the carboxyl group of RA. Therefore, we developed RA probe by conjugating RA and biotin moieties through a propyl linker. After assessing that RA‐biotin still retained the ability to effectively trigger HMGB1 release and T cell activation (Figure 5B,C; Figure S6A, Supporting Information), we incubated RA‐biotin with B16‐cell lysates and the precipitated proteins were subjected to sodium dodecyl sulfate‐polyacrylamide gel electrophoresis (SDS‐PAGE) and mass spectrometry (MS) analysis. As indicated in Figure 5D, top 10 candidate proteins were identified by the proteomic approach. We then validated RA interactors by HMGB1‐Gluc assay. Interestingly, silencing TDP‐43 by specific siRNA, but not other proteins identified by MS, significantly decreased RA‐induced HMGB1‐Gluc activity (Figure S6B, Supporting Information), indicating TDP‐43 was a RA‐interacting protein. Furthermore, endogenous affinity pull‐down assay confirmed the binding of RA with TDP‐43 in MC38 and B16 cell lysates (Figure 5E; Figure S6C, Supporting Information). Consistently, RA‐biotin‐captured TDP‐43 (Figure 5F) and RA‐induced HMGB1‐Gluc activity (Figure S6D, Supporting Information) were dramatically reduced in CRISPR‐Cas9‐engineered TDP‐43^KO^ tumor cells. These data suggest that RA selectively binds to TDP‐43 or a complex that contains TDP‐43.

To examine whether RA binds to TDP‐43 directly, we generated recombinant His‐TDP‐43 protein for further analysis. The purified His‐TDP‐43 was incubated with DMSO or increasing concentrations of RA‐biotin, and then the mixtures were resolved by IB with anti‐His antibody. As shown in Figure 5G, His‐TDP‐43 was pulled down by RA‐biotin in a concentration‐dependent manner. However, His‐TDP‐43 was not precipitated in the presence of excess free RA (Figure 5H), suggesting that there is a strong interaction between RA and recombinant TDP‐43. To further confirm RA directly binds to TDP‐43, we determined the target engagement of RA by using cellular thermal shift assay (CETSA).^[^ ^29^, ^30^ ^]^ Treatment of MC38 and B16 cells with RA led to significant thermal stabilization of TDP‐43 protein (Figure 5I,J), demonstrating the intracellular binding between RA and TDP‐43. Furthermore, surface plasmon resonance (SPR) assay also showed that RA highly bound to TDP‐43 and the determined equilibrium dissociation constant (KD) was about 1.3 µmol L^−1^ (Figure 5K). However, the KD of OA, an inactive RA analogue, toward TDP‐43 was dramatically increased to 76.12 µmol L^−1^ (Figure S6E, Supporting Information). We next conducted molecular dynamics analysis using the crystal structure of TDP‐43 (PDB ID: 4BS2) to investigate the possible binding modes of TDP‐43 with RA. Based on the 3D binding mode, we found RA could dock to the RNA recognition motifs (RRM) domain of TDP‐43 (Figure S6F, Supporting Information) that provides nuclear localization of TDP‐43. Particularly, RA forms favorable hydrogen bond interactions with residues including Arg197, Ala228, His256, and Ser258 (Figure 5L). To identify the residues most critical for RA binding, we individually mutated several of these interacting residues in TDP‐43, including K181A, R197A, A228G, H256A, and S258A. We found that A228G and H256A mutants dramatically reduced the ability of TDP‐43 binding of RA (Figure 5M). Moreover, TDP‐43^A228G^ and TDP‐43^H256A^, but not TDP‐43^WT^, could not restore RA‐triggered HMGB1‐Gluc activity when it was reintroduced into TDP‐43^KO^ cells (Figure 5N). These data illustrated that RA directly binds to TDP‐43 at Ala228 and His256. Collectively, these findings consistently reveal that TDP‐43 is a specific target for the action of RA.

### 2.6 Raddeanin A Triggers mtDNA Leakage into the Cytoplasm to Activate cGAS/STING Signaling

**Figure 6:**
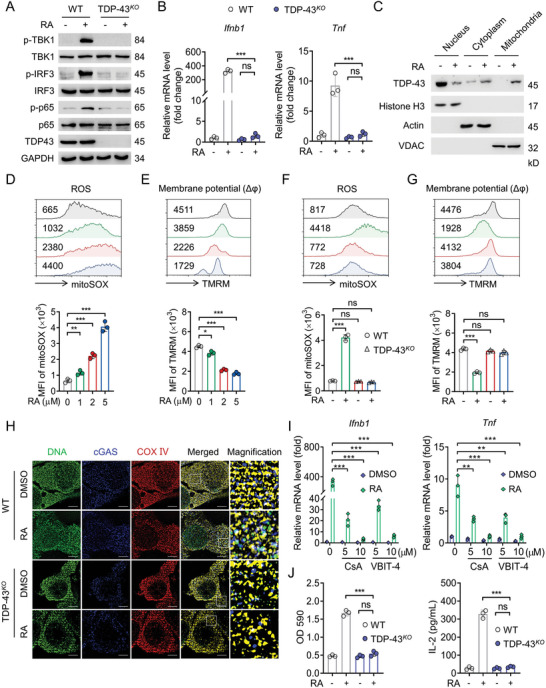
RA triggers mtDNA leakage into the cytoplasm to activate cGAS/STING signaling. A) IB analysis of the phosphorylation levels of TBK1, IRF3, and p65 in WT or TDP‐43^KO^ MC38 cells after RA (5 µM) treatment for 24 h. B) qPCR analysis of the expressions of Ifnb1 and Tnf in WT or TDP‐43^KO^ MC38 cells after RA (5 µM) treatment for 24 h. C) IB analysis determining the distribution of TDP‐43 in nucleus, cytoplasm, and mitochondria of MC38 cells treated with RA (5 µM) for 24 h. H3, Actin, and VDAC were used as the loading control for nucleus, cytoplasm, and mitochondria, respectively. D) ‐FACS analysis of the changes of ROS level (stained by mitoSOX) and E) membrane potential (mΔ*φ*, stained by TMRM) in MC38 cells treated with indicated dose of RA for 6 h. WT or TDP‐43^KO^ MC38 cells were treated with indicated RA for 6 h, the F) changes of ROS level and G) membrane potential were determined by FACS. H) WT or TDP‐43^KO^ MC38 cells were treated with RA (5 µM) for 12 h, and the co‐localization of cGAS and mtDNA was investigated by confocal microscopy. Scale bar: 5 µm. I) MC38 cells were pretreated with mtDNA leakage inhibitors CsA and VBIT‐4 for 2 h, followed by RA (5 µM) treatment for 24 h, the expressions of Ifnb1 and Tnf were analyzed by qPCR. J) WT or TDP‐43^KO^ B16 cells were treated with DMSO or RA (5 µM) for 20 h, followed by coculturing with BMDCs and B3Z cells for an additional 24 h, then LacZ activity and IL‐2 production were determined. Data are presented as mean ± SD, ^*^ *p* < 0.05, ^**^ *p* < 0.01, ^***^ *p* < 0.001, ns, not significant, D,E) one‐way ANOVA test, B,F,G,I,J) two‐way ANOVA test.

We next presumed that TDP‐43 might contribute to RA‐induced upregulation of cGAS/STING signaling. As expected, RA‐induced activation of signaling molecules downstream of cGAS/STING, including the phosphorylation of TBK1, IRF3, and p65, were greatly abolished in TDP‐43^KO^ MC38 (**Figure** **6**A) and B16 cells (Figure S7A, Supporting Information). Meanwhile, RA‐triggered Ifnb1 and Tnf expressions were also dramatically reduced by TDP‐43 depletion (Figure 6B; Figure S7B, Supporting Information). Given TDP‐43 is capable of shuttling between the nucleus and cytosol, we next investigated the cellular distribution of TDP‐43 after RA treatment. Cellular fraction assay revealed that RA treatment significantly increased the enrichment of TDP‐43 in the mitochondria (Figure 6C; Figure S7C, Supporting Information). We thus speculated that RA might cause mitochondrial destabilization in tumor cells. Indeed, the hallmarks of mitochondrial destabilization, such as increased reactive oxygen species (ROS) level and loss of membrane potential (mΔ*φ*), were clearly observed in RA‐treated MC38 (Figure 6D,E) and B16 cells (Figure S7D,E, Supporting Information). Additionally, depletion of TDP‐43 prevented RA‐mediated mitochondrial destabilization (Figure 6F,G; Figure S7F,G, Supporting Information). Taking an independent cellular immunofluorescence (IF) approach, we found RA treatment increased the co‐localization between cytoplasm mtDNA and cGAS, but depletion of TDP‐43 failed to do so (Figure 6H; Figure S7H, Supporting Information). These observations indicate RA treatment cause mitochondrial destabilization and mtDNA leakage. In agreement, disrupting mtDNA leakage by pharmacological inhibitors cyclosporin A (CsA)^[^ ^31^ ^]^ and VBIT‐4^[^ ^32^ ^]^ prevented RA‐mediated Ifnb1 and Tnf expressions in MC38 cells (Figure 6I), but not in TDP‐43‐depleted MC38 cells (Figure S7I, Supporting Information). Consistent with these results, RA treatment on TDP‐43^KO^ B16‐OVA cells could not further activate LacZ activity and IL‐2 release in B3Z cells (Figure 6J). All together, these findings potentially suggest RA bound to TDP‐43 triggers mitochondria destabilization and mtDNA leakage into the cytoplasm, thus activates cGAS/STING signaling.

### 2.7 The Combination of Raddeanin A and Anti‐Programmed Death 1 Effectively Suppresses Tumor Growth

**Figure 7:**
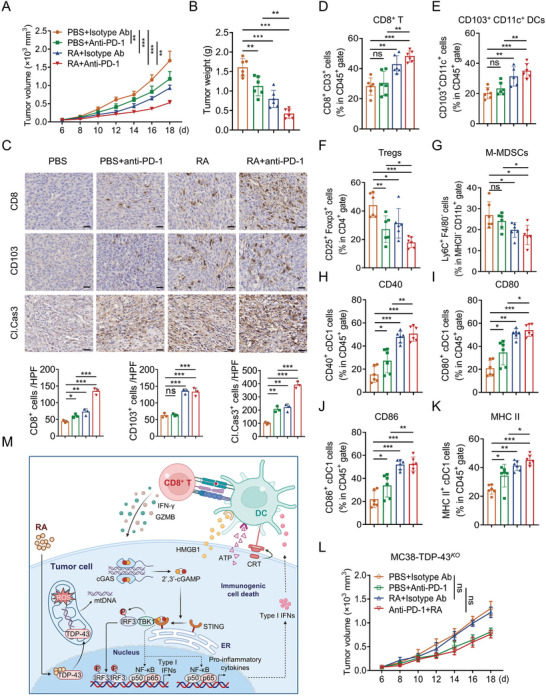
RA induces immune cell infiltration and potentiates efficacy of anti‐PD1 therapy. C57BL/6 mice bearing MC38 tumor were treated with PBS, anti‐PD‐L1 (100 µg per mouse), RA (4 mg kg^−1^), or the combination. A) Tumor volume and B) tumor weight were monitored. C) Representative IHC staining results for CD8, CD103, and cleaved caspase 3 in PBS, anti‐PD‐L1, RA, or the combination‐treated mice (Scale bar, 50 µm). Quantification of IHC staining is shown. FACS analyzing the populations of D) CD8^+^ T cells, E) cDC1, F) Tregs, G) M‐MDSCs, and surface expressions of H) CD40, I) CD80, J) CD86, and K) MHC‐II in cDC1 from PBS, anti‐PD‐1, RA, or the combination‐treated MC38 tumor. *n* = 6 mice per group. L) Tumor growth of TDP‐43^KO^ MC38 from PBS, anti‐PD1, RA, or the combination treated C57BL/6 mice (*n* = 6). *n* = 6 mice per group. Data are presented as mean ± SD, ^*^ *p* < 0.05; ^**^ *p* < 0.01, ^***^ *p* < 0.001, ns, no significant. M) Proposed model of RA eliciting tumor immunogenicity and enhancing antitumor immunity. This figure was created with BioRender.com. RA specifically bound to TDP‐43 and induced its localization to mitochondria and mtDNA release, leading to cGAS/STING‐dependent activation of NF‐*κ*B and type I IFN signaling, thereby reprogrammed TME. Data are presented as mean ± SD, ^*^ *p* < 0.05, ^**^ *p* < 0.01, ^***^ *p* < 0.001, ns, not significant, B–K) one‐way ANOVA test, A,L) two‐way ANOVA test.

As RA increased the PD‐L1 surface expression on tumor cells (Figure S8A,B, Supporting Information), and increased PD‐L1 abundance in tumors improves the efficacy of anti‐PD‐1 immunotherapy,^[^ ^33^, ^34^ ^]^ we therefore investigated whether combination treatments of RA and anti‐PD‐1 have synergistic anti‐tumor effects in the MC38 tumor model. To this aim, mice bearing MC38 were treated with PBS, RA, anti‐PD‐1 antibody, or the combination (Figure S8C, Supporting Information). We found that while RA or anti‐PD‐1 alone both partially reduced mouse tumor burden from control, combination treatments of RA with PD‐1 antibody achieved the best tumor growth inhibition (**Figure** **7**A,B; Figure S8D, Supporting Information). In addition, immunohistochemistry (IHC) assay (Figure 7C) and flow cytometry (FCM) analysis (Figure 7D,E) revealed that RA and anti‐PD‐1 antibody combination led to higher proportions of tumor‐infiltrating CD8^+^ T cells in TILs and CD103^+^ CD11c^+^ cDC1 cells than either therapy did alone. Moreover, a significant increase in cleaved caspase 3 level was found in RA and anti‐PD‐1 combination treatment group (Figure 7C), indicating RA and anti‐PD‐1 combination treatment induced an obvious apoptosis of tumor cells in vivo. Consistently, RA and anti‐PD‐1 combination treatment led to a remarkable increase in effector molecules including IFN‐*γ* and GZMB production by tumor‐infiltrating CD8^+^ T cells compared to that in each treatment alone (Figure S8E,F, Supporting Information), reinforcing that the cytotoxic CD8^+^ T cell activity was increased after RA and anti‐PD‐1combination treatment.

We next investigated the effect of RA on TME. Macrophage, myeloid‐derived suppressor cells (MDSCs) and regulatory T cells (Tregs) are the major immunosuppressive populations within TME.^[^ ^35^ ^]^ FCM analysis revealed that while the M1 macrophage, M2 macrophage, and polymorphonuclear MDSC (PMN‐MDSC) populations were not affected by RA and anti‐PD‐1 combination treatment (Figure S8G–I, Supporting Information), however, a significant decrease of Tregs and monocytic MDSC (M‐MDSCs) was observed within the tumor tissue in response to RA and anti‐PD‐1 combination treatment (Figure 7F,G). Furthermore, MC38‐tumor‐infiltrating cDC1 also showed upregulated CD40, CD80, CD86, and MHC‐II expression after RA and anti‐PD‐1 combination treatment (Figure 7H–K). These findings suggested that RA potentiated the antitumor efficacy of anti‐PD‐1 by reprograming the immune microenvironment. Importantly, RA along or combined with anti‐PD‐1 antibody failed to suppress the tumor growth of mice bearing TDP‐43^KO^ MC38 cells (Figure 7L), indicating that the tumor growth inhibition mediated by RA is mainly due to TDP‐43‐dependent regulation. Overall, these findings suggest that RA can be combined with anti‐PD‐1 antibody to improve its therapeutic effects.

## 3 Discussion

A determining step in the successful anticancer immune response is eliciting immunogenic antigen in tumors, which subsequent increases antigen presentation of DCs and activates T cell‐mediated adaptive immune response.^[^ ^36^ ^]^ Here, we discovered that RA is a potent ICD inducer possessing the capacity to induce antitumor adaptive immunity. We provided clear evidences that RA activates antigen‐specific immune responses and enhances immunotherapy responses by eliciting tumor cell ICD via targeting TDP‐43 (Figure 7M). Our findings thus illustrated a complex role of RA on both cancer cells and the TME that potentiates the efficacy of ICT.

Previous studies have reported that RA efficiently suppresses proliferation, invasion and induction of apoptosis in various human carcinogenic cells through multiple cell signaling pathways, including Wnt/*β*‐catenin, JAK‐STAT, and PI3‐AKT signaling,^[^ ^13^, ^37^ ^]^ however, whether RA may functions as an immune modulator and mediates its anti‐tumor efficacy remains unknown. In this study, we demonstrated that DCs‐mediated T cell immunity actually dominate RA's anti‐tumor activity. RA potentially elicited tumor cell ICD and induced effective T cells activation and boosted the production of IFN‐*γ* and GZMB by tumor‐infiltrating CD8^+^ T cells, indicating that RA mobilizes T cell immunity for tis anti‐tumor effect. It is well documented that DCs uptake and cross‐present tumor antigens to prime and activate cytotoxic CD8^+^ T lymphocytes for successful anticancer immune response,^[^ ^38^ ^]^ therefore, reactivating DCs in TME is considered as an ideal therapeutic strategy against cancer. Our results demonstrated that RA not only fosters the “adjuvanticity” of cancer cells by eliciting secretion or exposure of certain DAMPs, but also promotes DCs maturation and CD8^+^ T cell activation for tumor control. DCs can be subdivided into conventional DCs (cDCs, including CD8*α* ^+^/or CD103^+^ cDC1 subset, CD11b^+^ cDC2 subset), plasmacytoid DCs (pDCs, CD11^−^ B220^+^), and monocyte‐derived DCs (MoDCs, CD14^+^ CD206^+^).^[^ ^39^ ^]^ cDC1 and cDC2 subsets contribute to antitumor immunity through initiating and cross‐presenting antigens to CD8^+^ T or CD4^+^ T cells, respectively.^[^ ^40^ ^]^ pDCs and MoDCs show relatively poor priming of naïve T cells and play a vital role in innate antiviral immunity via producing IFN.^[^ ^21^ ^]^ Among them, cDC1 DCs (i.e., CD103^+^ CD11c^+^ DCs) are the major tumor antigen‐presenting cells in vivo for CD8^+^ T cells priming. Consistently, we found RA‐treated tumors displayed higher levels of infiltrating CD103^+^ CD11c^+^ DCs than the control group, and RA failed to suppress the tumor growth in cDC1‐depleted mice, suggesting a critical role of cDC1 in immune recognition and elimination of tumor. In support of this notion, other studies also shows that tumor cell‐derived, cDC1‐dependent T cell infiltration contributes to antitumor immunity in vivo.^[^ ^41^, ^42^ ^]^ We speculated that RA might induce a component shift of MoDCs (CD14^+^ CD206^+^) and pDCs (CD11^−^ B220^+^) to cDC1s (CD103^+^ CD11^+^ DCs) in TME. Further studies are warranted to validate whether RA may reprogram DCs in TME and instigate a functional differentiation of the DCs subpopulations.

The antitumor potential of RA can be mediated through the modulation of cell cycle and DNA damage in tumor cells.^[^ ^13^ ^]^ However, many chemotherapeutic drugs inducing cell cycle inhibition and DNA damage, such as oleanonic acid and temozolomide, did not induce tumor cell ICD in our and other's assays,^[^ ^43^ ^]^ suggesting that other effectors were involved in RA‐induced ICD. Our findings indicate that RA treatment significantly enriches NF‐*κ*B and type I IFN pathways in tumor cells, which is consistent with recent findings that tumor cell ICD can be induced via activation of the NF‐*κ*B and type I IFN signaling.^[^ ^44^, ^45^ ^]^ NF‐*κ*B signaling was required for tumor immunogenicity via regulating necroptosis and controlling IFN‐*β* expression in tumor cells.^[^ ^11^, ^46^ ^]^ Type I IFN signaling not only increases the immunogenicity of tumor cells through facilitating antigen presentation, but also reprogram the TME by recruiting and activating DCs and T cells.^[^ ^5^, ^47^ ^]^ Furthermore, cGAS/STING has been specifically implicated in driving the activation of NF‐*k*B and type I IFN pathways to enhance antitumor T cell responses. In line with this, our in‐depth analysis revealed that RA‐induced NF‐*κ*B and type I IFN activation in tumor cells depended on the cGAS/STING as silence of cGAS or STING, but not RIG‐1, MDA‐5, or TLR3, abolished NF‐*κ*B and type I IFN activation. Our findings thus suggest that tumor‐intrinsic STING/NF‐*κ*B and type I IFN signaling is critical for RA‐mediated tumor inhibition, and highlight a complex effect of RA on both cancer cells and the TME.

An important finding in this study is that RA triggers cGAS/STING‐mediated anticancer immunity via targeting TDP‐43. TDP‐43 plays crucial role in human cancer through regulation of the oncogene or tumor suppressor mRNA splicing and stability.^[^ ^48^ ^]^ Aberrant overexpression of TDP‐43 has been implicated in the pathogenesis of several types of human malignancies and tends to correlate with poor prognosis.^[^ ^49^ ^]^ Meanwhile, the cytoplasmic aggregation of TDP‐43 has been observed in neurons of ALS and FTD patients.^[^ ^50^ ^]^ Our findings revealed that RA specifically binds to TDP‐43, leading to mitochondrial TDP‐43 localization. In addition, RA‐induced leakage of mtDNA was associated with opening of the mPTP. Cytoplasmic DNA is sensed by intracellular DNA sensors such as cGAS/STING which activates TBK1 and IRF3 to potentiate antitumor immunity.^[^ ^51^ ^]^ Interestingly, depletion of cGAS/STING, but not the RNA sensor PKR or TLR3, abolished RA‐induced activation of the NF‐*κ*B and type I IFN signaling. Moreover, our results confirm that RA‐induced mitochondrial TDP‐43 localization drives cGAS/STING activation. TDP‐43 entry into mitochondria appears to be highly conserved evolutionarily from yeast to mice.^[^ ^52^, ^53^ ^]^ In line with our conclusion, recent studies demonstrate that mitochondrial localization of TDP‐43 resulted in mtDNA leakage into the cytoplasm and subsequent cGAS‐STING activation,^[^ ^17^, ^54^ ^]^ suggesting that targeting of mitochondrial localization of TDP‐43 is an alternative approach to enhancing ICT. Furthermore, targeting TDP‐43 is the key mechanism of RA‐treated tumor cell‐induced DCs activation as knockout of TDP‐43 in tumor cells greatly blunted the effect of RA on DCs. Therefore, TDP‐43 is an attractive target to effectively activate DCs for the development of potent immunotherapy. However, it is possible that RA may cause toxicity effect on the immune cells in TME owing to the wide distributed TDP‐43, hence, targeting delivery of RA to tumor site by nanoparticles or antibody‐drug conjugate strategies would help to elucidate this issue.

The immunosuppressive microenvironment is a major obstacle for successful tumor immunotherapy.^[^ ^35^ ^]^ To further validate tumor cell‐intrinsic mechanisms for enhancing ICT agent responsiveness, we explored tumor immune cell profiles including T cells, DCs, Tregs and MDSCs in mice treated with RA or anti‐PD‐L1. M1 macrophages inhibit tumor growth by secreting inhibitory factors and phagocytosis, whereas M2 macrophages facilitate tumor progression.^[^ ^55^ ^]^ M‐MDSCs and PMN‐MDSCs, the main subpopulations of MDSCs, inhibit tumor‐reactive T and NK cells via upregulation of PD‐L1 and arginase (ARG)‐1.^[^ ^56^ ^]^ Tregs suppress the antitumor immune response driven by CD8^+^ T cells by producing inhibitory cytokines and consumption of IL‐2.^[^ ^57^ ^]^ In addition to demonstrating RA and anti‐PD‐1 combination treatment activating CD8^+^ T cell for tumor eradication by increasing the antigen presentation of DCs, our data also indicated that RA and anti‐PD‐1 combination treatment activated T cell indirectly through alleviating M‐MDSCs and Tregs accumulation in the TME. These findings are consistent with other studies, showing that M‐MDSCs and Tregs trigger immunosuppressive microenvironment and promote tumor progression.^[^ ^58^ ^]^ It is well established tumor‐intrinsic ICD activated DCs and T cells, but the precise functional relationship between ICD and Tregs, MDSCs is unknown. It will be interesting to determine how RA‐elicited ICD inhibits the function of MDSCs and Tregs, thus expanding the T cell immune response, and whether TDP‐43 is involved.

In summary, our findings suggest that RA elicits tumor immunogenicity and enhances antitumor immunity by a DCs‐T cell‐dependent function via targeting TDP‐43. RA specifically bound to TDP‐43 and induced its mitochondria localization and mtDNA release, leading to cGAS/STING‐dependent activation of NF‐*κ*B and type I IFN signaling, thereby reprogrammed TME and sensitized tumor response to anti‐PD1 antibody treatment. Our findings provide a rationale for the potential application of combinatorial therapies of RA with ICT as promising and efficacious anticancer immunotherapies.

## 4 Experimental Section

### Cell Lines, Chemicals, Antibodies

Mouse melanoma cell B16, mouse colon adenocarcinoma cell MC38, and 293T cell lines were obtained from Institute of Basic Medicine, Chinese Academy of Medical Sciences (Beijing, China). B16‐OVA and MC38‐OVA were generated from B16 and MC38 respectively which were stably transfected with the plasmid pCI‐neo‐mOVA (Addgene). Mouse hybridoma cell B3Z was kindly gifted by Dr Nilabh Shastri (University of California, Berkeley, California, USA). All cell lines were cultured in PRMI 1640 (Gibco, Pittsburgh, PA, USA) or DMEM (Gibco, Pittsburgh, PA, USA) supplemented with 10% FBS, 0.1% streptomycin and 100 U mL^−1^ penicillin at 37 °C in a humidified atmosphere with 5% CO_2_. Natural product chemical monomers were purchased from selleck (Beijing, China). Other reagents and commercial assay kits used in this study were listed in Table S1, Supporting Information. The antibodies used in this study were listed in Tables S2 and S3, Supporting Information.

### Isolation and Culture of Bone Marrow‐Derived Dendritic Cells

Bone marrow (BM) cells were harvested from six‐to‐eight weeks old female C57BL/6J mice by flushing mouse femurs and tibias with RPMI 1640 medium containing 10% FBS and then filtered through a 70 µm strainer. After centrifugation, BM cells were resuspended in RBC lysis buffer (150 mm NH_4_Cl/10 mm NaHCO_3_/1 mm EDTA) for 5 min to remove red blood cells. The remaining BM cells were cultured in RPMI 1640 medium containing 10% FBS supplemented with 0.1% streptomycin, 100 U mL^−1^ penicillin, 50 µM 2‐mercaptoethanol, 50 ng mL^−1^ mGM‐CSF (Peprotech) and 20 ng mL^−1^ mIL‐4 (Peprotech), and the media were renewed every 3 days. After 7 days, most BM cells (>90%) were differentiated to DCs.

### Depletions of Dendritic Cells In Vivo

For DCs elimination in vivo, Cyt c (Sigma‐Aldrich) was administrated to mice by i.v. (5 mg per mouse in 100 µL PBS) at day ‐1, 7, and 14. Depletion of CD11c^+^ DCs in spleen and tumor tissues was verified by FCM.^[^ ^22^ ^]^

### Measurement of Dendritic Cells and T Cell Activation

To measure DCs activation and antigen presentation, B16‐OVA or MC38‐OVA cells seeded in 6‐well plates were treated with RA for 20 h before being co‐cultured with BMDCs for an additional 24 h. After that, co‐cultured cells were harvested and stained with fluorescence‐labeled antibodies against CD11c, CD40, CD86, CD80, MHC I, MHC II, and H2K^b^‐SIINFEKL. Stained cells were subsequently quantitatively analyzed by guava easyCyte flow cytometer with guavaSoft 3.1.

For T cell activation assay, B16‐OVA cells seeded in 96‐well plates were treated with RA for 20 h, and then co‐cultured with BMDCs and B3Z cells at a ratio of 1:1:5 for an additional 24 h. Co‐cultured cells in 96‐well plate were centrifuged at 400 g for 5 min at room temperature, and the culture supernatant was collected for ELISA to detect IL‐2 or IFN‐*γ*. T cells were stained with fluorescence‐labeled surface marker antibodies against CD8 and CD69. After that, cells were fixed and permeabilized with Cell Stimulation Cocktail (eBioscience, San Diego, CA, USA) and labeled with anti‐mouse IFN‐*γ* and GZMB. For LacZ reporter assay, B3Z cells in 96‐well culture plate were lysed in lysis buffer (250 mm Tris (pH 8.0), 0.1% Triton X‐100) (50 µL per well). After three cycles of freeze‐thaw, 50 µL of PBS containing 0.5% BSA and 100 µL of 1 mg mL^−1^ chlorophenolred *β*‐D‐galactopyranoside solution were added to each well and incubated at 37 °C for 12–18 h until adequate color development was achieved. Microtiter plate reader was used to measure color intensity at 580 nm.

### Detection of Surface Calreticulin, High‐Mobility Group Box 1‐Gluc Activity and Release of Adenosine Triphosphate

For measurement of HMGB1‐Gluc activity, B16 or MC38 cells stably transfected with HMGB1‐Gluc were seeded in 96‐well plate for 24 h, and then treated with indicated concentrations of RA for 20 h. The culture supernatant was gathered, and HMGB1‐Gluc activity was determined with Renilla Luciferase Reporter Assay (Promega). With similar experimental treatment, extracellular ATP in culture supernatant was measured by using the ENLITEN ATP Assay System (Promega). For detection of surface CRT, RA‐treated tumor cells were collected and washed twice with PBS before they were incubated with CRT mAb or IgG mAb Isotype Control conjugated with Alexa Fluor 488. Surface CRT was then quantified and analyzed by FCM.

### RNA Sequencing

DMSO or RA treated MC38 cells were gathered and total RNA was isolated using Trizol (ThermoFisher Scientific) according to the manufacturer's instructions. Total RNA was submitted to OE Biotech Co., Ltd. (Shanghai, China) for RNA‐seq using HiSeq X ten (Illumina). Raw data were screened using Trimmomatic to obtain the clean reads and were mapped to the Mus musculus genome (mm10/GRCm38) using HISAT.

### Quantitative Real‐Time PCR

Total RNA was extracted using RNeasy Kit (Qiagen) as manufacturer's instructions. Reverse transcription was conducted using a SuperScript III CellsDirect cDNA Synthesis Kit (Invitrogen). qPCR was carried out using TB Green Premix Ex Taq (Tli RNaseH Plus) (Takara). Relative mRNA expression was determined by the ΔΔCt method and normalized to GAPDH. Primers were designed using the Primer3Plus online tool and listed in the Table S4, Supporting Information.

### Synthesis of Raddeanin A‐Biotin and Raddeanin A‐Propanol

For the preparation of RA‐Biotin, to a solution of compound (+)‐Biotin *N*‐succinimidyl ester (500 mg, 1.46 mmol) in DMF (20 mL) was added anhydrous potassium carbonate (2.02 g, 14.6 mmol) in an ice bath. After stirring for 20 min, compound 3‐bromopropylamine hydrobromide (3.17 g, 14.6 mmol) was added. The mixture continued to be stirred until the starting material disappeared completely. The resulting precipitate was filtrated and the solvent was removed under vacuum. The resulting crude was purified by chromatography on silica gel to give compound I as a white foam solid. To a solution of compound RA (100 mg, 0.11 mmol) in DMF (2 mL) was added anhydrous potassium carbonate (46.2 mg, 0.33 mmol) at room temperature. After stirring for 10 min, compound I (1.32 g, 4.21 mmol) was added. Then the mixture continued to be stirred at room temperature until the starting material disappeared completely. The resulting precipitate was filtrated and the solvent was removed under vacuum. The resulting crude was purified by chromatography on silica gel to give compound RA‐Biotin as a white solid. The structure of RA‐Biotin was confirmed by NMR and MS.

For the synthesis of RA‐Propanol, to a solution of compound RA (30 mg, 0.033 mmol) in DMF (1.5 mL) was added anhydrous potassium carbonate (14 mg, 0.10 mmol) in an ice bath. After stirring for 10 min, compound 3‐bromopropan‐1‐ol (14 mg, 0.10 mmol) was added. The mixture continued to be stirred until the starting material disappeared completely. The resulting precipitate was filtrated and the solvent was removed under vacuum. The residue was subjected to chromatography on silica gel to give compound RA‐Propanol as a white solid. The structure of RA‐Propanol was certified by NMR and MS.

### Raddeanin A‐Biotin Pulldown Assay and Site‐Directed Mutagenesis

Streptavidin‐agarose beads and RA‐Biotin were incubated for 2 h at 25 °C in HEPES buffer (20 mmol L^−1^ HEPES, pH 7.4, 1 mmol L^−1^ dithiothreitol, 100 mmol L^−1^ NaCl, 0.05% NP‐40). His‐TDP‐43 protein, or B16, MC38 lysates, or 293T cell lysates expressed hemagglutinin (HA)‐TDP‐43‐wild type (WT), HA‐TDP43‐ K181A, R197A, A228G, H256A, S258A were preincubated with DMSO or various concentrations of RA at 25 °C for 2 h, and then incubated with RA‐Biotin‐streptavidin complex at 4 °C overnight. Following HEPES buffer wash, the proteins that had attached to the beads were analyzed by IB using His, HA, or TDP‐43 antibody. Site‐mutant plasmids of TDP‐43 were obtained by total gene synthesis and confirmed by sequencing (DIA‐UP Biotech, Beijing, China).

### Enzyme‐Linked Immunosorbent Assay

Levels of IL‐2, IFN‐*γ*, TNF‐*α*, IFN‐*β*, and CXCL10 in cell culture supernatants were measured by ELISA kits (R&D Systems, SM2000, MIF00, MTA00B, MIFNB0, DY466‐05) according to the manufacturer's instructions.

### Immunohistochemistry

Standard IHC staining in mouse tumor tissues was conducted as follows. Briefly, mouse tumor tissues were harvested and incubated with primary antibodies against CD8, CD103, and Cleaved Caspase3 at 4 °C overnight. HRP‐labeled secondary antibodies were then added and DAB was used as chromogenic reagent.

### Immunofluorescence

For IF, the cells were fixed in 4% formaldehyde for 15 min at room temperature, rinsed three times in PBS, and permeabilized for 10 min with PBS (0.5% Triton X‐100). The cells were then blocked with blocking Buffer (CST) for 60 min at RT, followed by incubation with primary antibodies against cGAS and DNA at 4 °C overnight. Following three rinses with PBS and 1 h incubation with secondary antibodies (goat anti‐mouse AF488 and goat anti‐rabbit AF405) at RT, cells were incubated with anti‐COX IV‐AF647 antibody at 4 °C overnight, and examined using confocal laser scanning microscope (Olympus, FV3000). The images were processed with Olympus cellSens software.

### Expression and Purification of Recombinant Transactive Responsive DNA‐Binding Protein 43 Protein

Full length of mouse TDP‐43 cDNA was cloned into pET‐22b (+) via XhoI and NdeI restriction sites. The plasmid was then transformed into *Escherichia coli* strain BL21 (DE3), and induced with 0.5 mm iospropyl *β*‐D‐thiogalactoside at 25 °C for 12 h. Cells were harvested by centrifugation and resuspended in 20 mm Tris‐HCl, 200 mm NaCl, pH 8.0 (buffer A) containing 1 µg mL^−1^ DNase I and 0.1 mg mL^−1^ lysozyme. After thawing, cells were ruptured by ultra‐sonication, and the resulting lysate was cleared by centrifugation (75 000 g for 20 min). The supernatant was applied onto a nickel chelating affinity column equilibrated in buffer A and eluted by an imidazole gradient. This was followed by size‐exclusion chromatography using a buffer of 20 mm Tris‐HCl, pH 7.5, 150 mm NaCl, and 1% glycerol on a Superdex 200 16/60 column (GE Healthcare). TDP‐43‐containing fractions were combined and concentrated for further use.

### Cellular Thermal Shift Assay

MC38 and B16 cells were harvested and lysed in lysis buffer (20 mM Tris‐HCl (pH 7.6), 250 mM NaCl, 3 mM EDTA, 3 mM EGTA, and 0.5% NP40) supplemented with protease inhibitors. To the supernatants, 20 µM RA or DMSO was added and incubated at 25 °C for 30 min. Cell lysates were then distributed into 8 different 200 µL PCR tubes with 100 µL of cell lysates in each tube. PCR tubes were then heated at their designated temperature (39–60 °C) for 3 min in the 96‐well thermal cycler. After that, samples were centrifuged at 20 000 g for 20 min at 4 °C, and the supernatants were analyzed by Western blot. All experiments were carried out in triplicate.^[^ ^59^, ^60^ ^]^

### Subcellular Fractionation

For isolation of intact mitochondria, nucleus, and cytoplasmic components, Minutetm Mitochondria Isolation Kit (Invent Biotechnologies) was used following manufacturer's instructions. Subcellular fractions were subsequently processed for Western blotting analysis.

### Detection of Mitochondrial Stress

MC38‐OVA or B16‐OVA cells were seeded in 6‐well plate for 24 h, and then treated with different concentrations of RA for 6–8 h. Treated cells were subsequently washed with PBS and incubated with MitoSOXTM Red (mitochondrial ROS production) or TMRM dye (mitochondrial membrane potential Δ*φm*) for 30 min at 37 °C away from light. Stained cells were then gently washed with PBS three times followed by resuspension in FACS buffer (PBS containing 1% FBS) and analyzed by FCM.

### Tumor Models and Treatments

6–8 weeks old female C57BL/6J mice and BALB/c nude mice were purchased from Beijing HFK Bio‐Technology.co., LTD (Beijing, China). All the mice were housed in specific pathogen‐free conditions, and all procedures were performed under guidelines approved by the animal ethics committee of the Institute of Medicinal Biotechnology, Chinese Academy of Medical Sciences (approval ethical numbers IMB‐20211109D201, IMB‐20211202D202, IMB‐20220217D204, IMB‐20220310D205, and IMB‐20220822D208). For in vivo study, MC38 tumor cells (1 × 10^6^ cells) were inoculated subcutaneously into the right flank of C57BL/6J mice or BALB/c nude mice. After 7 days, RA or vehicle was administrated by i.t. injection or by i.p. (1, 2, 4 mg kg^−1^) four times at the indicated time points. Anti‐PD‐1 was administrated by i.p. (100 µg per mouse, once every other day) three times. Anti‐CD8*α* and isotype depletion antibodies were given on day ‐1, 4, and 8 by i.p. (100 µg per mice). Tumor growth and body weight of mice was monitored and recorded every two days. Tumor volume was calculated as *π*/6 × tumor length × (tumor width)^2^. At the end of the experiments, the mice were sacrificed, and tumor tissues were separated for FCM analysis of tumor‐infiltrating immune cells. For the immunization study, 2 × 10^6^ MC38 cells, either treated with 20 µM RA or freeze‐thawed 3 times with liquid nitrogen, were inoculated subcutaneously into the right flank of C57BL/6J mice. One million live MC38 cells were injected into the opposing side after the tumors had grown for seven days. The tumor growth was then monitored.

### Isolation and Flow Cytometry Analysis of Tumor‐Infiltrating Lymphocytes

To generate single‐cell suspensions, tumor tissues were collected and cut into small pieces, and then strained through 70 µm cell strainers. For T cell analysis, cells were stained with CD45, CD3, CD8, IFN‐*γ*, and GZMB. For DCs analysis, cells were marked with CD45, CD11c, CD103, MHC II, CD80, CD86, and CD40. For Tregs analysis, cells were labeled with CD45, CD3, CD4, CD25, and Foxp3. For MDSCs analysis, cells were stained with CD45, CD11b, MHC II, F4/80, Ly6C (M‐MDSC), and Ly6G (PMN‐MDSC). For Macrophage analysis, cells were marked with CD45, CD11b, F4/80, MHC II (M1), and CD206 (M2). Stained cells were subsequently quantitatively analyzed by guava easyCyte flow cytometer with guavaSoft 3.1.

### CRISPR/Cas9‐Mediated Knockout and RNA Interference

CHOPCHOP (http://chopchop.cbu.uib.no/) was used to design sgRNAs, which were then cloned into LentiCRISPRv2. 293T cells in 10 cm Petri dishes were transfected with 5 µg lentiviral plasmid, 5 µg psPAX2, and 1 µg VSVG. Lentivirus‐containing media were gathered at 72 h after transfection and strained through a 0.45 µm filter. B16 or MC38 cells were infected with lentivirus for 24 h, and then refreshed with media containing puromycin. sgRNAs sequences were presented in Table S5, Supporting Information.

siRNAs were designed on siDESIGN Center (https://horizondiscovery.com/) and cloned into the pSEB‐HUS plasmid. For retrovirus production, the plasmids were transfected into 293T cells along with the plasmid pCL‐Ampho. The supernatants containing retrovirus were collected and used to infect B16 cells. The stably infected B16 cells were selected and maintained using Blasticidin S (InvivoGen, USA). siRNAs for target genes were listed in Table S6, Supporting Information.

### Surface Plasmon Resonance Analysis

The SPR experiments were carried out with the Reichert 2SPR system at room temperature. RA or OA was dissolved in 1 × PBST containing 1% DMSO. Recombinant mouse TDP‐43 was immobilized on SR7000 GOLD SENSOR SLIDE in 10 mm Sodium acetate buffer (pH 4.5). The remaining active sites were blocked with 1 mol L^−1^ ethanolamine (pH 8.5). Indicated concentrations of RA or OA were delivered into the system at a flow rate of 20 µL min^−1^ for 120 s. The binding kinetics were determined using SPRAutolink software.^[^ ^60^ ^]^

### Molecular Docking

Discovery Studio 4.5 and UCSF chimera1.7 were used to perform the molecular docking of RA with 3D structure of TDP‐43 (PDB code: 4BS2). The regularized protein was employed to identify the crucial amino acids in the anticipated binding site. The docked chemical was given a score based on its binding mode to the binding site.

### Statistics

Data are presented as mean ± SEM and evaluated using 2‐tailed unpaired Student's *t*‐test between two groups or one‐ or two‐way ANOVA followed by Bonferroni's post test between multiple groups. All graphs and statistical analyses were produced using GraphPad Prism 8. A *p*‐value of less than 0.05 was deemed significant.

## Conflict of Interest

The authors declare no conflict of interest.

## Author Contributions

M.Y. and J.D. contributed equally to this work. H.D. and M.Y. conceived and designed this study; M.Y. conducted most experiments and data analysis; J.D., X.L., L.L., Z.K., and N.Z. performed parts of the experiments; Z.L., D.X., and X.Z. provided reagents, synthesized the RA‐biotin, and performed molecular docking; H.D. supervised the study and interpreted results, wrote, and revised the manuscript.

## Supporting information

Supporting Information

## Data Availability

The data that support the findings of this study are available from the corresponding author upon reasonable request.

## References

[1] S. C. Wei , C. R. Duffy , J. P. Allison , Cancer Discovery 2018, 8, 1069. 30115704 10.1158/2159-8290.CD-18-0367

[2] P. Sharma , B. A. Siddiqui , S. Anandhan , S. S. Yadav , S. K. Subudhi , J. Gao , S. Goswami , J. P. Allison , Cancer Discovery 2021, 11, 838. 33811120 10.1158/2159-8290.CD-20-1680

[3] R. Zappasodi , T. Merghoub , J. D. Wolchok , Cancer Cell 2018, 33, 581. 29634946 10.1016/j.ccell.2018.03.005 PMC5896787

[4] C. Pfirschke , C. Engblom , S. Rickelt , V. Cortez‐Retamozo , C. Garris , F. Pucci , T. Yamazaki , V. Poirier‐Colame , A. Newton , Y. Redouane , Y. J. Lin , G. Wojtkiewicz , Y. Iwamoto , M. Mino‐Kenudson , T. G. Huynh , R. O. Hynes , G. J. Freeman , G. Kroemer , L. Zitvogel , R. Weissleder , M. J. Pittet , Immunity 2016, 44, 343. 26872698 10.1016/j.immuni.2015.11.024 PMC4758865

[5] L. Galluzzi , A. Buque , O. Kepp , L. Zitvogel , G. Kroemer , Nat. Rev. Immunol. 2017, 17, 97. 27748397 10.1038/nri.2016.107

[6] D. M. S. Hossain , S. Javaid , M. Cai , C. Zhang , A. Sawant , M. Hinton , M. Sathe , J. Grein , W. Blumenschein , E. M. Pinheiro , A. Chackerian , J. Clin. Invest. 2018, 128, 644. 29337311 10.1172/JCI94586 PMC5785250

[7] L. Menger , E. Vacchelli , S. Adjemian , I. Martins , Y. Ma , S. Shen , T. Yamazaki , A. Q. Sukkurwala , M. Michaud , G. Mignot , F. Schlemmer , E. Sulpice , C. Locher , X. Gidrol , F. Ghiringhelli , N. Modjtahedi , L. Galluzzi , F. Andre , L. Zitvogel , O. Kepp , G. Kroemer , Sci. Transl. Med. 2012, 4, 143ra99. 10.1126/scitranslmed.3003807 22814852

[8] D. V. Krysko , A. D. Garg , A. Kaczmarek , O. Krysko , P. Agostinis , P. Vandenabeele , Nat. Rev. Cancer 2012, 12, 860. 23151605 10.1038/nrc3380

[9] M. Obeid , A. Tesniere , F. Ghiringhelli , G. M. Fimia , L. Apetoh , J. L. Perfettini , M. Castedo , G. Mignot , T. Panaretakis , N. Casares , D. Metivier , N. Larochette , P. van Endert , F. Ciccosanti , M. Piacentini , L. Zitvogel , G. Kroemer , Nat. Med. 2007, 13, 54. 17187072 10.1038/nm1523

[10] L. Apetoh , F. Ghiringhelli , A. Tesniere , A. Criollo , C. Ortiz , R. Lidereau , C. Mariette , N. Chaput , J. P. Mira , S. Delaloge , F. Andre , T. Tursz , G. Kroemer , L. Zitvogel , Immunol. Rev. 2007, 220, 47. 17979839 10.1111/j.1600-065X.2007.00573.x

[11] N. Yatim , H. Jusforgues‐Saklani , S. Orozco , O. Schulz , R. B. da Silva , C. R. e Sousa , D. R. Green , A. Oberst , M. L. Albert , Science 2015, 350, 328. 26405229 10.1126/science.aad0395 PMC4651449

[12] A. Sistigu , T. Yamazaki , E. Vacchelli , K. Chaba , D. P. Enot , J. Adam , I. Vitale , A. Goubar , E. E. Baracco , C. Remedios , L. Fend , D. Hannani , L. Aymeric , Y. Ma , M. Niso‐Santano , O. Kepp , J. L. Schultze , T. Tuting , F. Belardelli , L. Bracci , V. La Sorsa , G. Ziccheddu , P. Sestili , F. Urbani , M. Delorenzi , M. Lacroix‐Triki , V. Quidville , R. Conforti , J. P. Spano , L. Pusztai , et al., Nat. Med. 2014, 20, 1301. 25344738 10.1038/nm.3708

[13] I. Naz , S. Ramchandani , M. R. Khan , M. H. Yang , K. S. Ahn , Molecules 2020, 25, 1035, 32106609 10.3390/molecules25051035 PMC7179125

[14] M. Neumann , D. M. Sampathu , L. K. Kwong , A. C. Truax , M. C. Micsenyi , T. T. Chou , J. Bruce , T. Schuck , M. Grossman , C. M. Clark , L. F. McCluskey , B. L. Miller , E. Masliah , I. R. Mackenzie , H. Feldman , W. Feiden , H. A. Kretzschmar , J. Q. Trojanowski , V. M. Lee , Science 2006, 314, 130. 17023659 10.1126/science.1134108

[15] Y. M. Ayala , P. Zago , A. D'Ambrogio , Y. F. Xu , L. Petrucelli , E. Buratti , F. E. Baralle , J. Cell Sci. 2008, 121, 3778. 18957508 10.1242/jcs.038950

[16] P. H. Kuo , L. G. Doudeva , Y. T. Wang , C. K. Shen , H. S. Yuan , Nucleic Acids Res. 2009, 37, 1799. 19174564 10.1093/nar/gkp013 PMC2665213

[17] C. H. Yu , S. Davidson , C. R. Harapas , J. B. Hilton , M. J. Mlodzianoski , P. Laohamonthonkul , C. Louis , R. R. J. Low , J. Moecking , D. De Nardo , K. R. Balka , D. J. Calleja , F. Moghaddas , E. Ni , C. A. McLean , A. L. Samson , S. Tyebji , C. J. Tonkin , C. R. Bye , B. J. Turner , G. Pepin , M. P. Gantier , K. L. Rogers , K. McArthur , P. J. Crouch , S. L. Masters , Cell 2020, 183, 636. 33031745 10.1016/j.cell.2020.09.020 PMC7599077

[18] Z. Wang , J. Chen , J. Hu , H. Zhang , F. Xu , W. He , X. Wang , M. Li , W. Lu , G. Zeng , P. Zhou , P. Huang , S. Chen , W. Li , L. P. Xia , X. Xia , J. Clin. Invest. 2019, 129, 4850. 31408442 10.1172/JCI127471 PMC6819145

[19] S. Qian , Q. L. Chen , J. L. Guan , Y. Wu , Z. Y. Wang , Chem. Pharm. Bull. 2014, 62, 779. 10.1248/cpb.c14-00138 25087630

[20] X. Shen , L. Li , Y. He , X. Lv , J. Ma , Aging 2021, 13, 7166. 33621954 10.18632/aging.202574 PMC7993697

[21] M. Swiecki , M. Colonna , Nat. Rev. Immunol. 2015, 15, 471. 26160613 10.1038/nri3865 PMC4808588

[22] M. L. Lin , Y. Zhan , A. I. Proietto , S. Prato , L. Wu , W. R. Heath , J. A. Villadangos , A. M. Lew , Proc. Natl. Acad. Sci. U. S. A. 2008, 105, 3029. 18272486 10.1073/pnas.0712394105 PMC2268579

[23] L. Alexopoulou , A. C. Holt , R. Medzhitov , R. A. Flavell , Nature 2001, 413, 732. 11607032 10.1038/35099560

[24] H. Kato , O. Takeuchi , S. Sato , M. Yoneyama , M. Yamamoto , K. Matsui , S. Uematsu , A. Jung , T. Kawai , K. J. Ishii , O. Yamaguchi , K. Otsu , T. Tsujimura , C. S. Koh , C. R. e Sousa , Y. Matsuura , T. Fujita , S. Akira , Nature 2006, 441, 101. 16625202 10.1038/nature04734

[25] S. Liu , X. Cai , J. Wu , Q. Cong , X. Chen , T. Li , F. Du , J. Ren , Y. T. Wu , N. V. Grishin , Z. J. Chen , Science 2015, 347, aaa2630. 25636800 10.1126/science.aaa2630

[26] B. Lu , T. Nakamura , K. Inouye , J. Li , Y. Tang , P. Lundback , S. I. Valdes‐Ferrer , P. S. Olofsson , T. Kalb , J. Roth , Y. Zou , H. Erlandsson‐Harris , H. Yang , J. P. Ting , H. Wang , U. Andersson , D. J. Antoine , S. S. Chavan , G. S. Hotamisligil , K. J. Tracey , Nature 2012, 488, 670. 22801494 10.1038/nature11290 PMC4163918

[27] J. Vincent , C. Adura , P. Gao , A. Luz , L. Lama , Y. Asano , R. Okamoto , T. Imaeda , J. Aida , K. Rothamel , T. Gogakos , J. Steinberg , S. Reasoner , K. Aso , T. Tuschl , D. J. Patel , J. F. Glickman , M. Ascano , Nat. Commun. 2017, 8, 750. 28963528 10.1038/s41467-017-00833-9 PMC5622107

[28] S. M. Haag , M. F. Gulen , L. Reymond , A. Gibelin , L. Abrami , A. Decout , M. Heymann , F. G. van der Goot , G. Turcatti , R. Behrendt , A. Ablasser , Nature 2018, 559, 269. 29973723 10.1038/s41586-018-0287-8

[29] R. Jafari , H. Almqvist , H. Axelsson , M. Ignatushchenko , T. Lundback , P. Nordlund , D. M. Molina , Nat. Protoc. 2014, 9, 2100. 25101824 10.1038/nprot.2014.138

[30] F. B. Reinhard , D. Eberhard , T. Werner , H. Franken , D. Childs , C. Doce , M. F. Savitski , W. Huber , M. Bantscheff , M. M. Savitski , G. Drewes , Nat. Methods 2015, 12, 1129. 26524241 10.1038/nmeth.3652

[31] A. M. McGee , C. P. Baines , Biochem. J. 2012, 443, 185. 22236255 10.1042/BJ20111881 PMC3508683

[32] J. Kim , R. Gupta , L. P. Blanco , S. Yang , A. Shteinfer‐Kuzmine , K. Wang , J. Zhu , H. E. Yoon , X. Wang , M. Kerkhofs , H. Kang , A. L. Brown , S. J. Park , X. Xu , E. Z. van Rilland , M. K. Kim , J. I. Cohen , M. J. Kaplan , V. Shoshan‐Barmatz , J. H. Chung , Science 2019, 366, 1531. 31857488 10.1126/science.aav4011 PMC8325171

[33] J. Zhang , X. Bu , H. Wang , Y. Zhu , Y. Geng , N. T. Nihira , Y. Tan , Y. Ci , F. Wu , X. Dai , J. Guo , Y. H. Huang , C. Fan , S. Ren , Y. Sun , G. J. Freeman , P. Sicinski , W. Wei , Nature 2018, 553, 91. 29160310 10.1038/nature25015 PMC5754234

[34] J. Haanen , Cell 2017, 170, 1055. 28886376 10.1016/j.cell.2017.08.031

[35] D. I. Gabrilovich , S. Ostrand‐Rosenberg , V. Bronte , Nat. Rev. Immunol. 2012, 12, 253. 22437938 10.1038/nri3175 PMC3587148

[36] D. S. Chen , I. Mellman , Immunity 2013, 39, 1. 23890059 10.1016/j.immuni.2013.07.012

[37] Q. Wang , J. Mo , C. Zhao , K. Huang , M. Feng , W. He , J. Wang , S. Chen , Z. Xie , J. Ma , S. Fan , Cell Death Dis. 2018, 9, 376. 29515110 10.1038/s41419-018-0417-0 PMC5841366

[38] S. K. Wculek , F. J. Cueto , A. M. Mujal , I. Melero , M. F. Krummel , D. Sancho , Nat. Rev. Immunol. 2020, 20, 7. 31467405 10.1038/s41577-019-0210-z

[39] A. T. Satpathy , X. Wu , J. C. Albring , K. M. Murphy , Nat. Immunol. 2012, 13, 1145. 23160217 10.1038/ni.2467 PMC3644874

[40] A. R. Sanchez‐Paulete , A. Teijeira , F. J. Cueto , S. Garasa , J. L. Perez‐Gracia , A. Sanchez‐Arraez , D. Sancho , I. Melero , Ann. Oncol. 2017, 28, xii44. 28945841 10.1093/annonc/mdx237

[41] J. Li , K. T. Byrne , F. Yan , T. Yamazoe , Z. Chen , T. Baslan , L. P. Richman , J. H. Lin , Y. H. Sun , A. J. Rech , D. Balli , C. A. Hay , Y. Sela , A. J. Merrell , S. M. Liudahl , N. Gordon , R. J. Norgard , S. Yuan , S. Yu , T. Chao , S. Ye , T. S. K. Eisinger‐Mathason , R. B. Faryabi , J. W. Tobias , S. W. Lowe , L. M. Coussens , E. J. Wherry , R. H. Vonderheide , B. Z. Stanger , Immunity 2018, 49, 178. 29958801 10.1016/j.immuni.2018.06.006 PMC6707727

[42] H. Liu , J. Golji , L. K. Brodeur , F. S. Chung , J. T. Chen , R. S. deBeaumont , C. P. Bullock , M. D. Jones , G. Kerr , L. Li , D. P. Rakiec , M. R. Schlabach , S. Sovath , J. D. Growney , R. A. Pagliarini , D. A. Ruddy , K. D. MacIsaac , J. M. Korn , E. R. McDonald III , Nat. Med. 2019, 25, 95. 30559422 10.1038/s41591-018-0302-5

[43] A. Q. Sukkurwala , S. Adjemian , L. Senovilla , M. Michaud , S. Spaggiari , E. Vacchelli , E. E. Baracco , L. Galluzzi , L. Zitvogel , O. Kepp , G. Kroemer , Oncoimmunology 2014, 3, e28473. 25050214 10.4161/onci.28473 PMC4063139

[44] J. Ahn , T. Xia , A. R. Capote , D. Betancourt , G. N. Barber , Cancer Cell 2018, 33, 862. 29706455 10.1016/j.ccell.2018.03.027 PMC6177226

[45] L. Deng , H. Liang , M. Xu , X. Yang , B. Burnette , A. Arina , X. D. Li , H. Mauceri , M. Beckett , T. Darga , X. Huang , T. F. Gajewski , Z. J. Chen , Y. X. Fu , R. R. Weichselbaum , Immunity 2014, 41, 843. 25517616 10.1016/j.immuni.2014.10.019 PMC5155593

[46] K. Taniguchi , M. Karin , Nat. Rev. Immunol. 2018, 18, 309. 29379212 10.1038/nri.2017.142

[47] S. R. Woo , L. Corrales , T. F. Gajewski , Annu. Rev. Immunol. 2015, 33, 445. 25622193 10.1146/annurev-immunol-032414-112043

[48] X. Chen , Z. Fan , W. McGee , M. Chen , R. Kong , P. Wen , T. Xiao , X. Chen , J. Liu , L. Zhu , R. Chen , J. Y. Wu , Protein Cell 2018, 9, 848. 28952053 10.1007/s13238-017-0480-9 PMC6160384

[49] X. Ma , Y. Ying , H. Xie , X. Liu , X. Wang , J. Li , Front. Oncol. 2021, 11, 755096. 34778070 10.3389/fonc.2021.755096 PMC8581290

[50] A. Prasad , V. Bharathi , V. Sivalingam , A. Girdhar , B. K. Patel , Front. Mol. Neurosci. 2019, 12, 25. 30837838 10.3389/fnmol.2019.00025 PMC6382748

[51] T. Sen , B. L. Rodriguez , L. Chen , C. M. D. Corte , N. Morikawa , J. Fujimoto , S. Cristea , T. Nguyen , L. Diao , L. Li , Y. Fan , Y. Yang , J. Wang , B. S. Glisson , Wistuba II , J. Sage , J. V. Heymach , D. L. Gibbons , L. A. Byers , Cancer Discovery 2019, 9, 646. 30777870 10.1158/2159-8290.CD-18-1020 PMC6563834

[52] L. Ruan , C. Zhou , E. Jin , A. Kucharavy , Y. Zhang , Z. Wen , L. Florens , R. Li , Nature 2017, 543, 443. 28241148 10.1038/nature21695 PMC5793917

[53] S. A. Davis , S. Itaman , C. M. Khalid‐Janney , J. A. Sherard , J. A. Dowell , N. J. Cairns , M. A. Gitcho , Neurosci. Lett. 2018, 678, 8. 29715546 10.1016/j.neulet.2018.04.053 PMC5975202

[54] H. Yong , S. Wang , F. Song , Liver Int. 2021, 41, 1969. 33830629 10.1111/liv.14895

[55] M. M. Kaneda , K. S. Messer , N. Ralainirina , H. Li , C. J. Leem , S. Gorjestani , G. Woo , A. V. Nguyen , C. C. Figueiredo , P. Foubert , M. C. Schmid , M. Pink , D. G. Winkler , M. Rausch , V. J. Palombella , J. Kutok , K. McGovern , K. A. Frazer , X. Wu , M. Karin , R. Sasik , E. E. Cohen , J. A. Varner , Nature 2016, 539, 437. 27642729 10.1038/nature19834 PMC5479689

[56] F. Veglia , M. Perego , D. Gabrilovich , Nat. Immunol. 2018, 19, 108. 29348500 10.1038/s41590-017-0022-x PMC5854158

[57] K. Itahashi , T. Irie , H. Nishikawa , Eur. J. Immunol. 2022, 52, 1216. 35879813 10.1002/eji.202149358

[58] C. Groth , X. Hu , R. Weber , V. Fleming , P. Altevogt , J. Utikal , V. Umansky , Br. J. Cancer 2019, 120, 16. 30413826 10.1038/s41416-018-0333-1 PMC6325125

[59] X. Liu , M. Yin , J. Dong , G. Mao , W. Min , Z. Kuang , P. Yang , L. Liu , N. Zhang , H. Deng , Acta Pharm. Sin. B 2021, 11, 3134. 34745852 10.1016/j.apsb.2021.03.039 PMC8551420

[60] Y. Liu , X. Liu , N. Zhang , M. Yin , J. Dong , Q. Zeng , G. Mao , D. Song , L. Liu , H. Deng , Acta Pharm. Sin. B 2020, 10, 2299. 33354502 10.1016/j.apsb.2020.06.014 PMC7745128

