# Supplementary material for: Raddeanin A Enhances Mitochondrial DNA‐cGAS/STING Axis‐Mediated Antitumor Immunity by Targeting Transactive Responsive DNA‐Binding Protein 43
Source: Adv Sci (Weinh). 2023 Mar 6;10(13):2206737. doi: 10.1002/advs.202206737 (PMC10161045; doi:10.1002/advs.202206737)
Supplement: Supplementary file 1 — Supporting Information [file ADVS-10-2206737-s001.pdf]

ADVANCED SCIENCE
Open Access

Supporting Information

for *Adv. Sci.*, DOI 10.1002/advs.202206737

Raddeanin A Enhances Mitochondrial DNA-cGAS/STING Axis-Mediated Antitumor Immunity by Targeting Transactive Responsive DNA-Binding Protein 43

Mingxiao Yin, Jingwen Dong, Cuicui Sun, Xiaojia Liu, Zhirui Liu, Lu Liu, Zean Kuang, Na Zhang, Dian Xiao, Xinbo Zhou and Hongbin Deng**

Supporting Information for

Raddeanin A enhances mitochondrial DNA-cGAS/STING axis-mediated antitumor immunity by targeting TDP-43

Mingxiao Yin, Jingwen Dong, Cuicui Sun, Xiaojia Liu, Zhirui Liu, Lu Liu, Zeang Kuang, Na Zhang, Dian Xiao, Xinbo Zhou, * *and Hongbin Deng* *

This file includes:

Figure S1. RA induces an apoptosis-dependent ICD.

Figure S2. RA-treated tumor cells promote DCs maturation.

Figure S3. RA suppresses tumor growth and shows little toxicity and side effect in mice.

Figure S4. RA activates DCs and $CD8^+$ T cells *in vivo*.

Figure S5. RA induces cGAS/STING-dependent NF-κB and type I IFN signaling activation in B16 cells.

Figure S6. TDP-43 is a specific target for RA.

Figure S7. RA induces mtDNA leakage and activates cGAS/STING signaling.

Figure S8. Effects of RA on tumor growth and tumor microenvironment.

Figure S9. Gating strategies for FASC analysis.

Table S1. Reagents and commercial assay kits used in this study.

Table S2. Antibodies used for immunoblotting.

Table S3. Antibodies used for FACS, IF and IHC analysis.

Table S4. Forward and reverse primers for qPCR.

Table S5. sgRNA sequence for knocking out the indicated proteins.

Table S6. siRNA sequence for knocking down the indicated proteins.

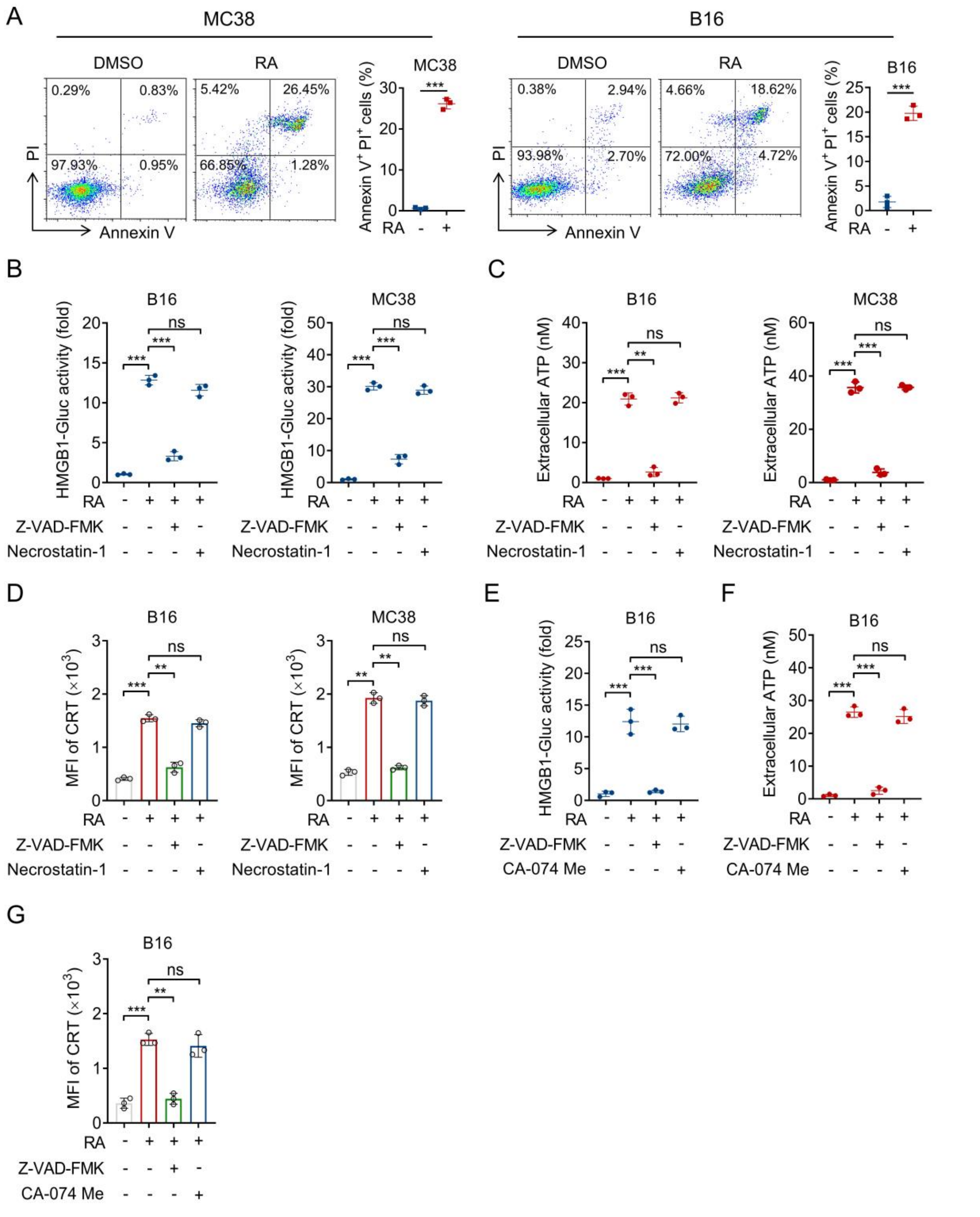

Figure S1. RA induces an apoptosis-dependent ICD. (A) FASC determining the apoptosis in B16 and MC38 cells treated with DMSO or RA (5 μM) for 20 hours. (B-D) B16 and MC38 cells were treated with RA (5 μM) alone, or together with Z-VAD-FMK (100 μM) or Necrostatin-1 (10 μM) for 20 hours, HMGB1-Gluc luciferase activities (B), extracellular ATP levels (C), and surface expressions of CRT (D) were measured. (E-G) B16 cells were treated with RA (5 μM) alone, or together with cathepsin B inhibitor CA-074 Me (50 μM) for 20 hours, HMGB1-Gluc luciferase activities (E), extracellular ATP levels (F), and surface expressions of CRT (G) were measured. Data are shown as mean ± SEM of 3 independent experiments. ** $P < 0.01$, *** $P < 0.001$, ns, not significant, unpaired Student's *t* test (A), two-way ANOVA test (B-G).

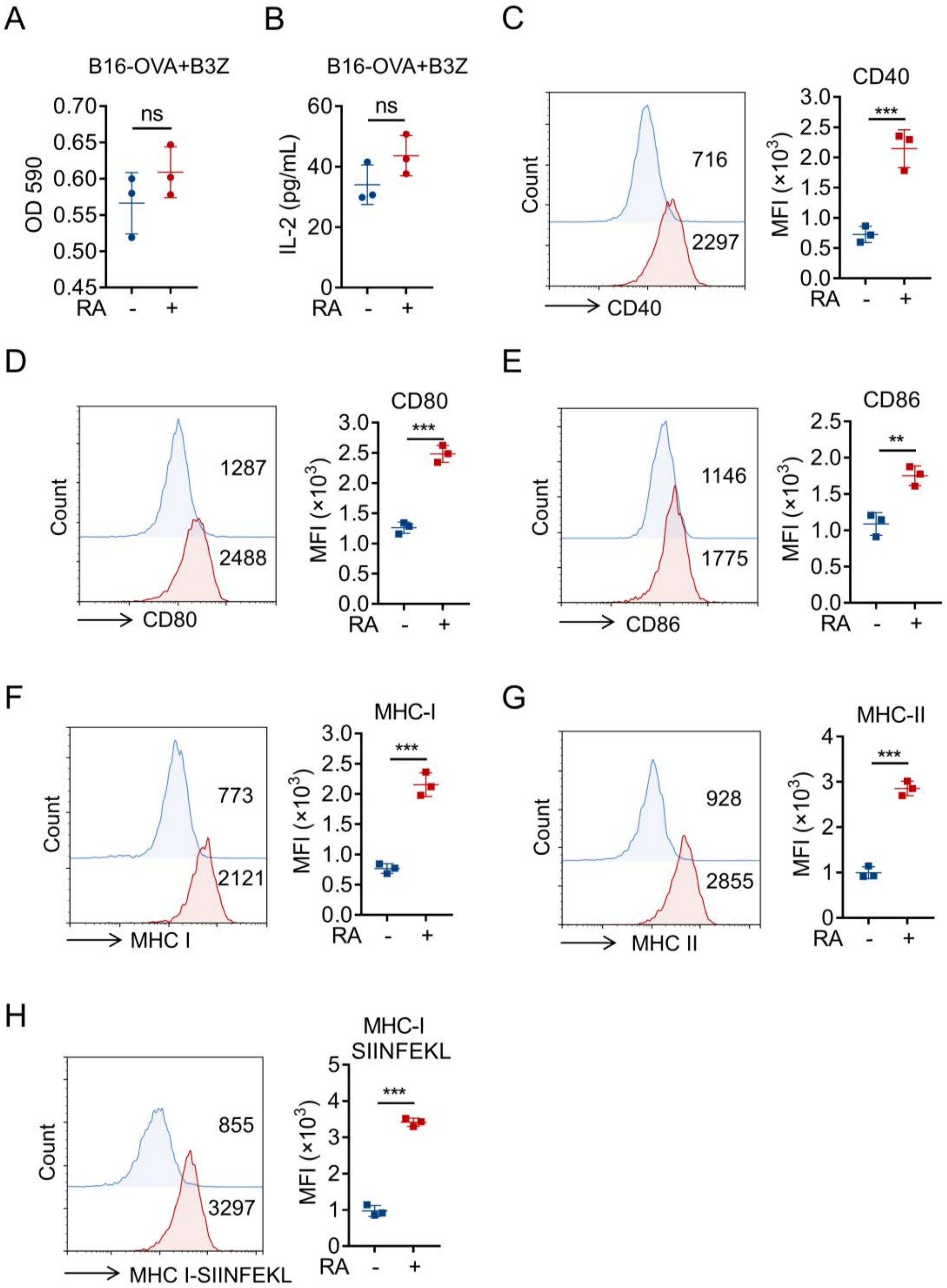

Figure S2. RA-treated tumor cells promote DCs maturation. (A and B) B16-OVA cells were treated with DMSO or RA (5 μM) for 24 hours, followed by coculturing with B3Z cells for an additional 24 hours, and then LacZ activity (A) and IL-2 production (B) were measured. (C-H) MC38-OVA cells were treated with DMSO or RA (5 μM) for 24 hours, then cocultured with BMDCs for an additional 24 hours, after which surface expressions of CD40, CD80, CD86, MHC-I, MHC-Ⅱ, and MHC-I-SⅡNFEKL on CD11c^{+} BMDCs were determined by FACS. Data are shown as mean ± SEM of 3 independent experiments. ** P < 0.01, *** P < 0.001, ns, not significant, unpaired Student's *t* test (A-H).

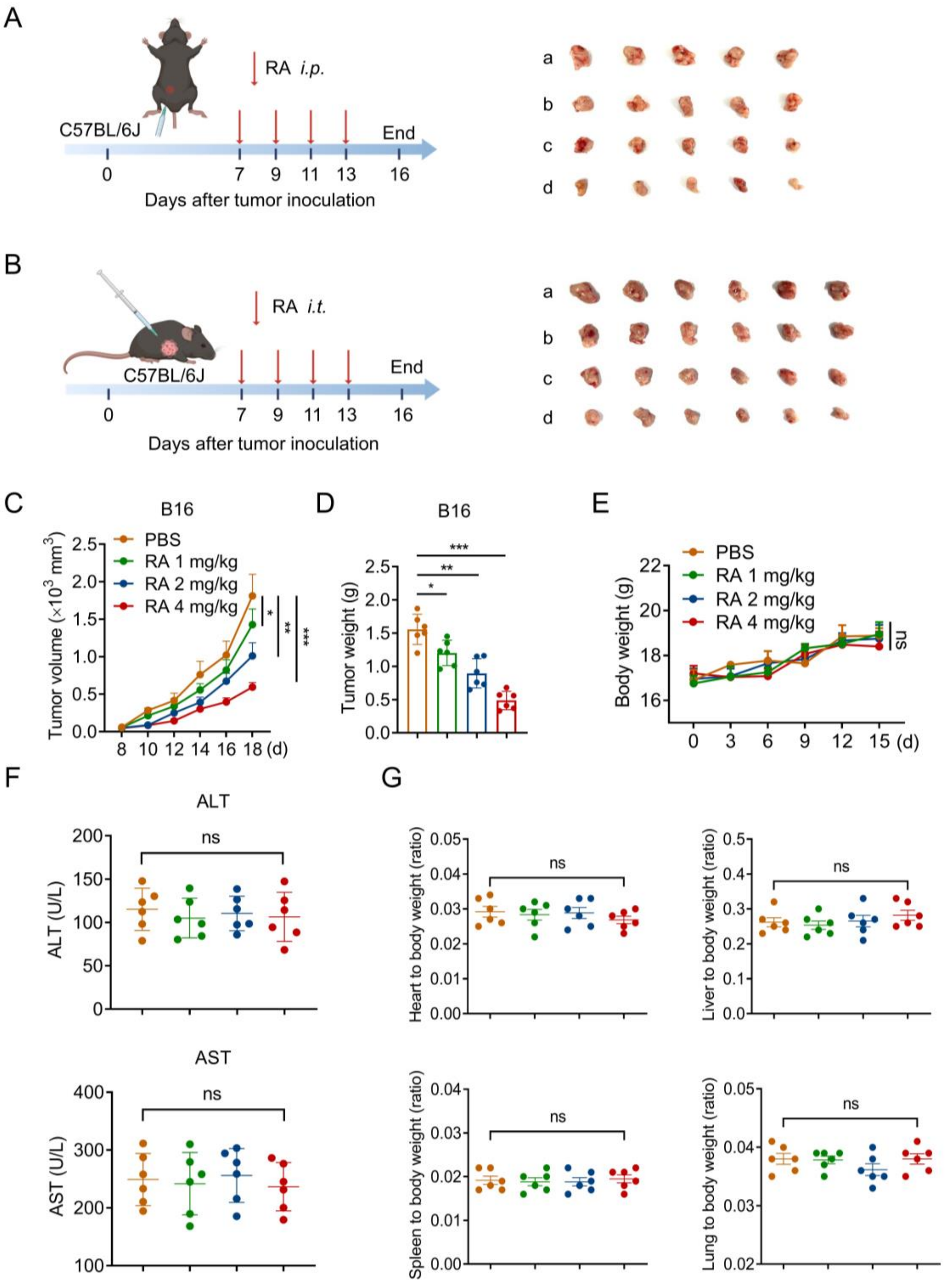

Figure S3. RA suppresses tumor growth and shows little toxicity and side effect in mice. (A-B) C57BL/6 mice with subcutaneous MC38 tumor were intraperitoneally (*i.p.*) or intratumorally (*i.t.*) injected with PBS or RA (1, 2 and 4 mg/kg) at the indicated times. The arrows indicate the treatment protocol. The tumors were *Ex vivo* observed from the PBS or RA treated mice. (C-D) C57BL/6 mice with subcutaneous B16 tumor were intratumorally (*i.t*) injected with PBS or RA (1, 2 and 4 mg/kg), the tumor volume (C) and tumor weight (D) were monitored. (E) The curves of the body weight of RA (4 mg/kg, *i.t.*) treated mice were recorded every three days. (F) The serum biochemistry indices ALT and AST of each group after RA (4 mg/kg, *i.t.*) treatment were measured. (G) After RA (4 mg/kg, *i.t*) treatment for 16 days, heart, liver, spleen, and lung of each group were taken out for weighing, the ratios of organ weight to body weight were shown. Data are presented as mean ± SD, n=6 per group; *

P < 0.05, ** P < 0.01, *** P < 0.001, ns, no significant, one-way ANOVA test (D, F, G), two-way ANOVA test (C, E).

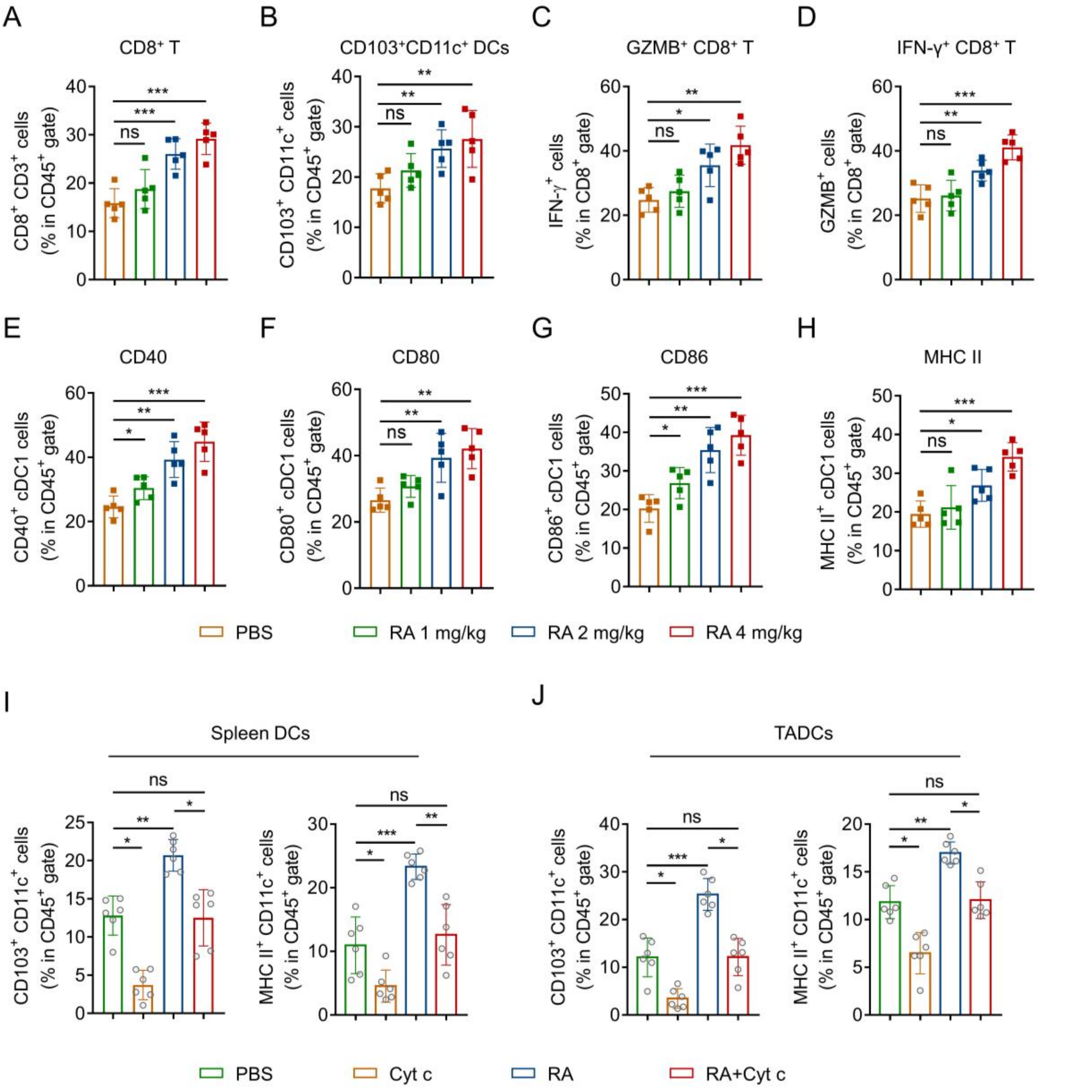

Figure S4. RA activates DCs and CD8$^+$ T cells *in vivo*. C57BL/6 mice with subcutaneous MC38 tumor were intraperitoneally (*i.p.*) injected with PBS or RA (1, 2 and 4 mg/kg). (A-D) FACS analyzing the numbers of tumor-infiltrating CD8$^+$ T cells (A), CD103$^+$ CD11c$^+$ DCs (B), and effector molecules GZMB (C) and IFN-γ (D) in CD8$^+$ T cells. (E-H) Surface expression levels of CD40, CD80, CD86, and MHC-Ⅱ on cDC1 cells were determined by FACS. n = 5 mice per group. (I and J) C57BL/6 mice bearing MC38 tumor were treated with vehicle, *Cyt c* that depleted DCs, and/or RA (4 mg/kg, *i.t.*). The populations of cDC1 and expression levels of MHC-Ⅱ on cDC1 cells in spleen and tumor were determined by FACS. n = 6 mice per group. Data are presented as mean ± SD, * P < 0.05, ** P < 0.01, *** P < 0.001, ns, not significant, one-way ANOVA test (A-J).

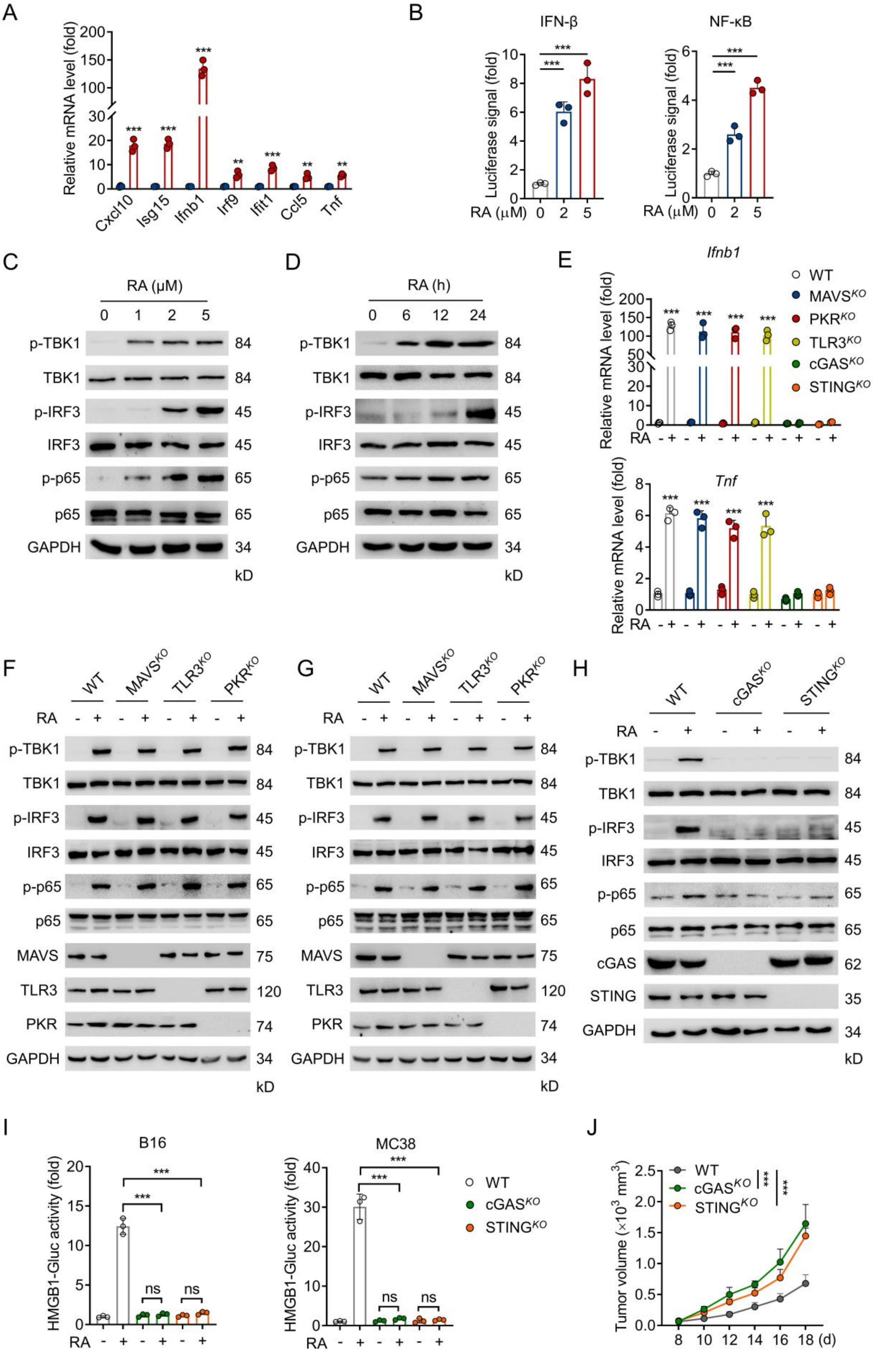

A
Relative mRNA level (fold)
Cxcl10
Isg15
Ifnb1
Irf9
Ifit1
Ccl5
Tnf
B
IFN-β
NF-κB
Luciferase signal (fold)
RA (μM) 0 2 5
C
RA (μM)
0 1 2 5
D
RA (h)
0 6 12 24
p-TBK1 84
TBK1 84
p-IRF3 45
IRF3 45
p-p65 65
p65 65
GAPDH 34
kD
E
Ifnb1
Tnf
WT
MAVSKO
PKRKO
TLR3KO
cGASKO
STINGKO
Relative mRNA level (fold)
RA
F
G
WT
MAVSKO
TLR3KO
PKRKO
RA
MAVS 75
TLR3 120
PKR 74
H
WT
cGASKO
STINGKO
cGAS 62
STING 35
I
B16
MC38
HMGB1-Gluc activity (fold)
ns
WT
cGASKO
STINGKO
J
Tumor volume (×10³ mm³)
WT
cGASKO
STINGKO
8 10 12 14 16 18 (d)

Figure S5. RA induces cGAS/STING-dependent NF-κB and type I IFN signaling activation in B16 cells. (A) qPCR analyzing NF-κB and type I IFN pathways associated genes in B16 cells treated with DMSO or RA (5 µM) for 24 hours. (B) Reporter gene assay determining the IFN-β and NF-κB activity in B16 cells treated with DMSO or RA (5 µM) for 24 hours. (C and D) Immunoblotting analysis of the phosphorylation levels of TBK1, IRF3 and p65 in B16 cells treated with indicated concentrations of RA for 24 hours (C), or with RA (5 µM) for different time (D). (E) Innate immune sensors in B16 cells were knockout by sgRNA, the expression of *Ifnb1* and *Tnf* were examined by qPCR after treatment with DMSO or RA (5 µM) for 24 hours. (F and G) Immunoblotting analysis of the phosphorylation levels of TBK1, IRF3 and p65 in WT, $MAVS^{KO}$, $TLR3^{KO}$, or PKR^{KO} MC38 cells (F) and B16 cells (G) treatment with DMSO or RA (5 µM) for 24 hours. (H) Immunoblotting analysis of the phosphorylation levels of TBK1, IRF3 and p65 in WT, $cGAS^{KO}$, or $STING^{KO}$ B16 cells treated with DMSO or RA (5 µM) for 24 hours. (I) Luciferase assay analysis of the HMGB1-Gluc activity in WT or $cGAS^{KO}$, $STING^{KO}$ B16-HMGB1-Gluc and MC38-HMGB1-Gluc cells after treatment with DMSO or RA (5 µM) for 24 hours. (J) Tumor growth of $cGAS^{KO}$ or $STING^{KO}$ MC38 from RA (4 mg/kg) treated C57BL/6 mice (n=6). Data are shown as mean ± SEM of 3 independent experiments. ** $P < 0.01$, *** $P < 0.001$, unpaired Student's *t* test (A), one-way ANOVA test (B), two-way ANOVA test (E, I, J).

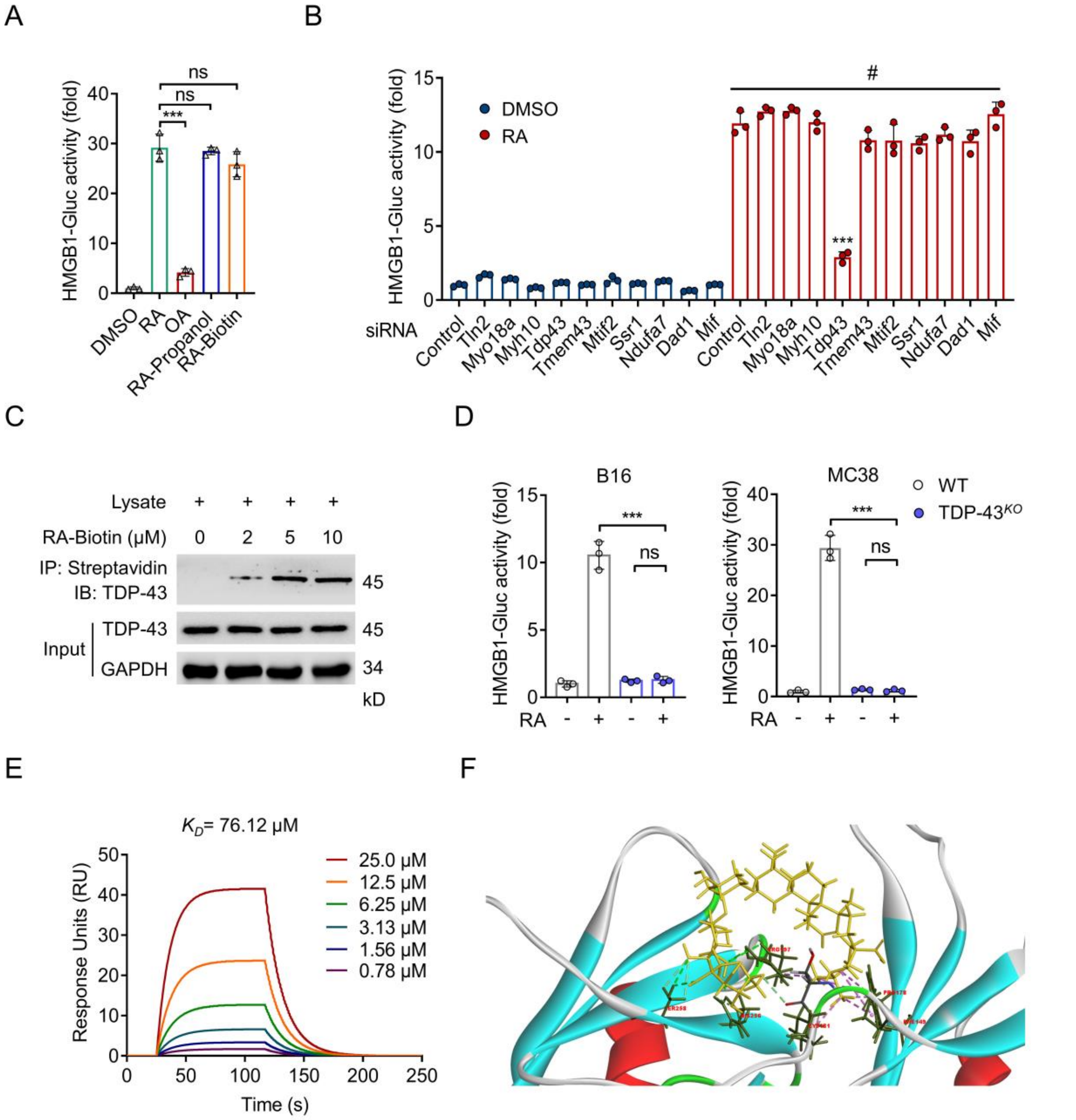

Figure S6. TDP-43 is a specific target for RA. (A) Luciferase assay determining the HMGB1 release in MC38-HMGB1-Gluc cells treated with indicated compounds for 20 hours. (B) Luciferase assay analyzing the HMGB1-Gluc activity in B16-HMGB1-Gluc cells with the indicated genes knockout after treatment with DMSO or RA (5 μM) for 20 hours. (C) B16 cell lysate was incubated with indicated RA-biotin overnight at 4 °C, the mixtures were precipitated by streptavidin-agarose and immunoblotted with TDP-43 antibody. (D) Luciferase assay analysis of the HMGB1-Gluc activity in WT or TDP-43^{KO} MC38-HMGB1-Gluc and B16-HMGB1-Gluc cells after treatment with DMSO or RA (5 μM) for 24 hours. (E) SPR analysis of the OA and TDP-43 binding. (F) Molecular docking model revealing RA binds to the RRM domain of TDP-43. Data are shown as mean ± SEM of 3 independent experiments. *** $P < 0.001$, $^{\#}$ $P < 0.001$ for RA versus DMSO (B), ns, not significant, one-way ANOVA test (A), two-way ANOVA test (B, D).

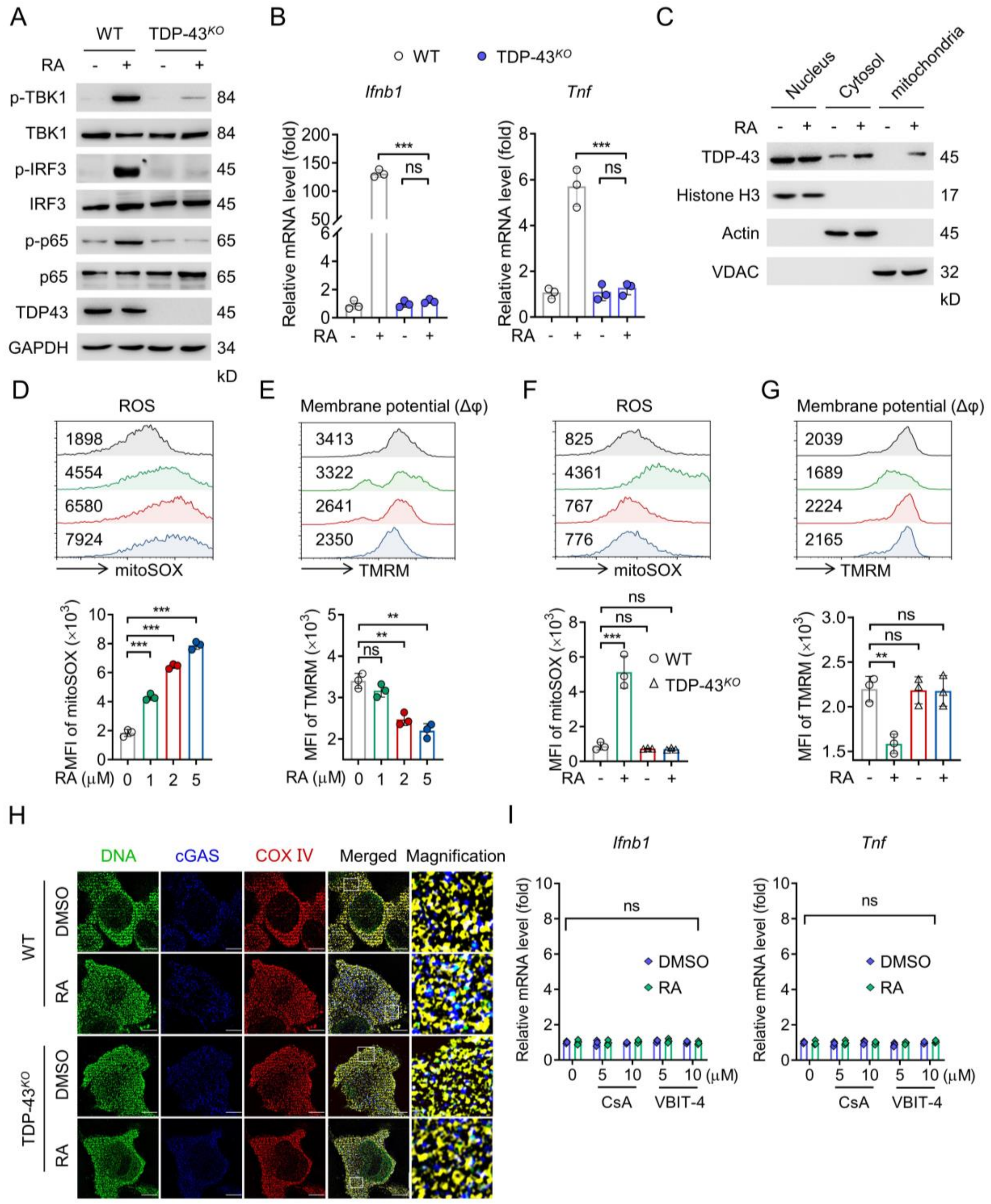

Figure S7. RA induces mtDNA leakage and activates cGAS/STING signaling. (A) Immunoblotting analysis of the phosphorylation levels of TBK1, IRF3 and p65 in WT or TDP-43^{KO} B16 cells after RA (5 μM) treatment for 24 hours. (B) qPCR analysis of the expressions of *Ifnb1* and *Tnf* in WT or TDP-43^{KO} B16 cells after RA (5 μM) treatment for 24 hours. (C) Immunoblotting determining the distribution of TDP-43 in nucleus, cytoplasm and mitochondria of B16 cells treated with RA (5 μM) for 24 hours. H3, actin, and VDAC were used as the loading control for nucleus, cytoplasm and mitochondria, respectively. (D and E) FASC analysis of the changes of ROS level (D) and membrane potential (mΔφ, E) in B16 cells treated with indicated dose of RA for 6 hours. (F and G) WT or TDP-43^{KO} B16 cells were treated with indicated RA for 6 hours, the changes of ROS level (F) and membrane

potential (G) were then determined by FASC. (H) WT or TDP-43^{KO} B16 cells were treated with RA (5 μM) for 12 hours, and the co-localization of cGAS and mtDNA was investigated by confocal microscopy. Scale bar: 5 μm. (I) TDP-43^{KO} B16 cells were pretreated with mtDNA leakage inhibitors CsA and VBIT-4, followed by RA treatment for 24 hours, the expressions of *Ifnb1* and *Tnf* were analyzed by qPCR. Data are shown as mean ± SEM of 3 independent experiments. ** P < 0.01, *** P < 0.001, ns, not significant, one-way ANOVA test (D, E), two-way ANOVA test (B, F, G, I).

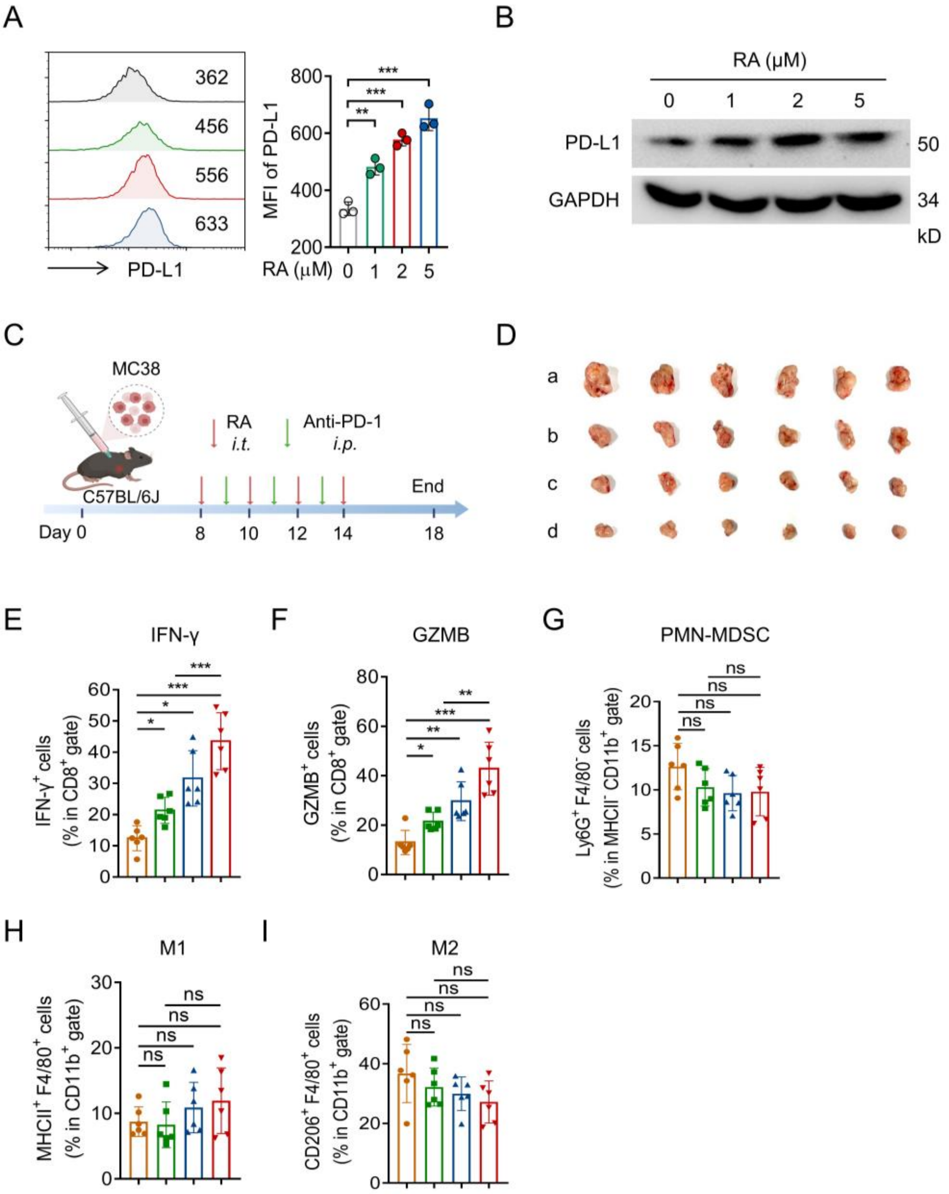

Figure S8. Effects of RA on tumor growth and tumor microenvironment. (A and B) FACS (A) and immunoblotting (B) analysis of surface PD-L1 levels in MC38 cells treated with indicated dose of RA for 24 hours. (C-D) C57BL/6 mice bearing MC38 tumor were treated with PBS, anti-PD1, RA or the combination. The arrows show the treatment protocol (C), and the MC38 tumors were *ex vivo* observed (D). (E-I) The levels of IFN-γ (E) and GzmB (F) secreted from CD8^{+}T cells, and populations of PMS-MDSC cells (G), M1 macrophages (H), and M2 macrophages (I) were analyzed by FASC. Data are shown as mean ± SEM of 3 independent experiments. * P < 0.05, ** P < 0.01, *** P < 0.001, ns, not significant, one-way ANOVA test (A, E-I).

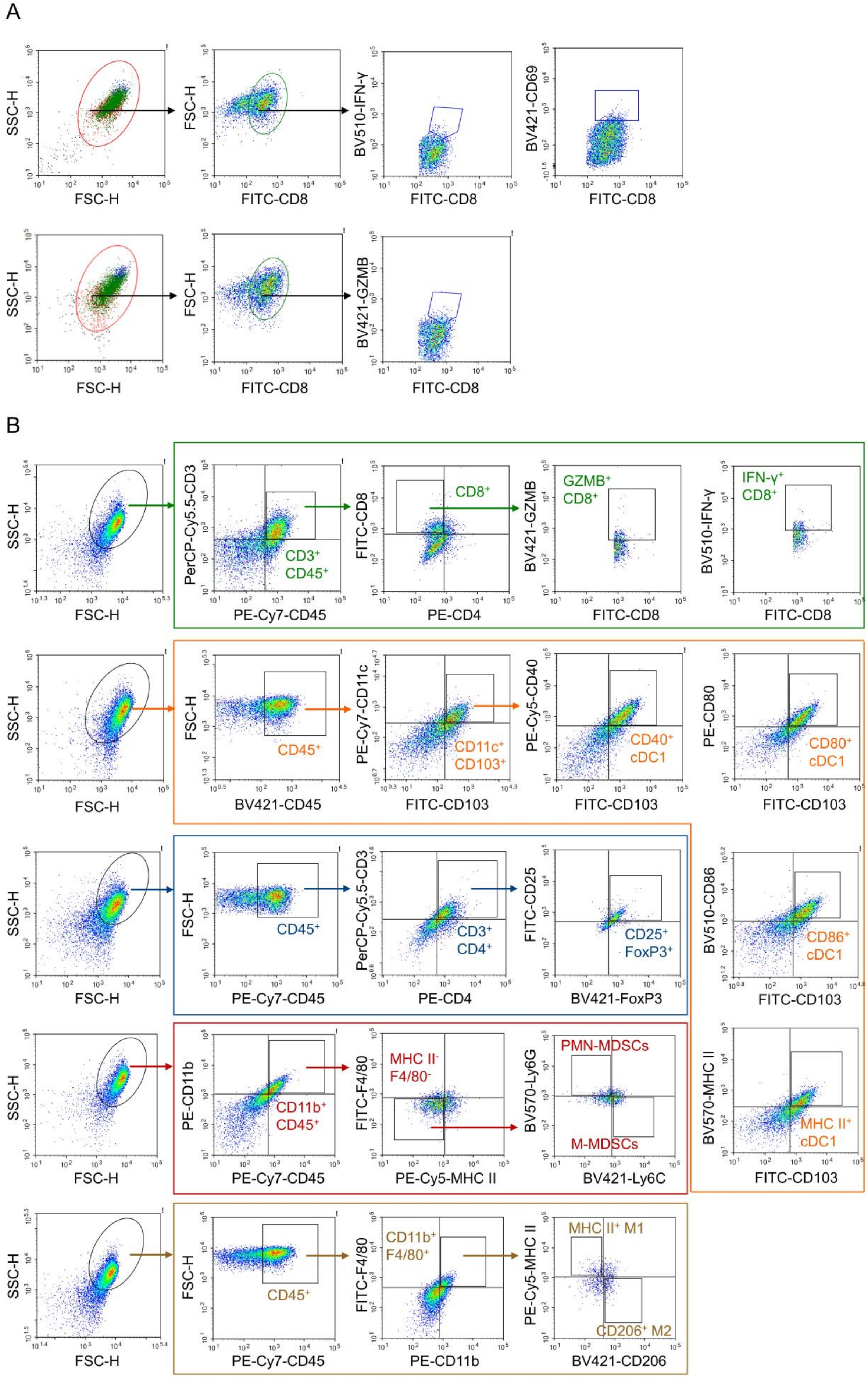
A
SSC-H
FSC-H
FITC-CD8
BV510-IFN-γ
BV421-CD69
BV421-GZMB
B
PerCP-Cy5.5-CD3
PE-Cy7-CD45
CD3+ CD45+
FITC-CD8
PE-CD4
CD8+
BV421-GZMB
GZMB+ CD8+
BV510-IFN-γ
IFN-γ+ CD8+
BV421-CD45
CD45+
PE-Cy7-CD11c
FITC-CD103
CD11c+ CD103+
PE-Cy5-CD40
CD40+ cDC1
PE-CD80
CD80+ cDC1
CD3+ CD4+
FITC-CD25
BV421-FoxP3
CD25+ FoxP3+
BV510-CD86
CD86+ cDC1
PE-CD11b
CD11b+ CD45+
FITC-F4/80
PE-Cy5-MHC II
MHC II- F4/80-
BV570-Ly6G
BV421-Ly6C
PMN-MDSCs
M-MDSCs
BV570-MHC II
MHC II+ cDC1
PE-CD11b
CD11b+ F4/80+
PE-Cy5-MHC II
BV421-CD206
MHC II+ M1
CD206+ M2

Figure S9. Gating strategies for FASC. (A) Gating strategy for $CD8^+$ T cell, CD69, IFN-γ and GZMB in tumor cells-BMDCs-T cells co-culture assay. (B) Flow cytometry gating strategies for analysis of tumor-infiltrating $CD8^+$ T, DCs, Tregs, MDSCs, and macrophages were shown.

Table S1. Reagents and commercial assay kits used in this study

**REAGENTS/COMMERCIAL KITS**	**SOURCE**	**IDENTIFIER**
Natural Product Library	Selleck	Cat# L1400
Oleanolic Acid	Selleck	Cat# S2334
H-151	Selleck	Cat# S6652
RU.521	Selleck	Cat# S6841
Cyclosporin A	Selleck	Cat# S2286
VBIT-4	Selleck	Cat# S3544
MitoSOXTM Red	Thermo Fisher Scientific	Cat# M36008
Tetramethylrhodamine methyl ester perchlorate (TMRM)	Thermo Fisher Scientific	Cat# T5428
Chlorophenolred *β*-D-galactopyranoside (CPRG)	Cayman Chemical	Cat# 29707
Cytochrome c	Sigma-Aldrich	Cat# C2506
Blocking buffer	Cell Signaling Technology	Cat# 12411
Renilla-Glo™ Luciferase Assay Kit	Promega	Cat# E2710
ENLITEN® ATP assay system	Promega	Cat# FF2000
RNeasy Kit	Qiagen	Cat# 75162
SuperScript™ III CellsDirect™ cDNA Synthesis Kit	Invitrogen	Cat# 18080300
TB Green® Premix Ex Taq™ (Tli RNaseH Plus)	Takara	Cat# RR420A
MinuteTM Mitochondria Isolation Kit	Invent Biotechnologies	Cat# MP-007
Mouse IL-2 Quantikine ELISA Kit	R&D Systems	Cat# SM2000
Mouse IFN-gamma Quantikine ELISA Kit	R&D Systems	Cat# MIF00

Mouse TNF-alpha Quantikine ELISA Kit	R&D Systems	Cat# MTA00B
Mouse IFN-beta Quantikine ELISA Kit	R&D Systems	Cat# MIFNB0
Mouse CXCL10/IP-10/CRG-2 ELISA Kit	R&D Systems	Cat# DY466-05
InVivoMAb anti-mouse PD-1 (CD279)	Bio X Cell	Cat# BE0033-2
InVivoMAb polyclonal Armenian hamster IgG	Bio X Cell	Cat# BE0091
InVivoMAb anti-mouse CD8α	Bio X Cell	Cat# BE0004-1
InVivoMAb rat IgG2a isotype control, anti-trinitrophenol	Bio X Cell	Cat# BE0089
Streptavidin (Sepharose® Bead Conjugate)	Cell Signaling Technology	Cat# 3419
SR7000 GOLD SENSOR SLIDE	Reichert Inc.	Cat# 13206066
Recombinant Murine GM-CSF	Peprotech	Cat# 315-03
Recombinant Murine IL-4	Peprotech	Cat# 214-14

Table S2. Antibodies used for immunoblotting.

**ANTIBODY**	**SOURCE**	**IDENTIFIER**
TDP43 Antibody	Cell Signaling Technology	Cat# 3448
GAPDH Rabbit mAb	Cell Signaling Technology	Cat# 5174
α-Tubulin Antibody	Cell Signaling Technology	Cat# 2144
β-Actin Mouse mAb	Cell Signaling Technology	Cat# 3700S
VDAC Rabbit mAb	Cell Signaling Technology	Cat# 4661
Histone H3 Rabbit mAb	Cell Signaling Technology	Cat# 4499
CGAS Rabbit mAb	Cell Signaling Technology	Cat# 31659
STING Rabbit mAb	Cell Signaling Technology	Cat# 13647
IRF-3 Rabbit mAb	Cell Signaling Technology	Cat# 4302
Phospho-IRF-3 (Ser396) Rabbit mAb	Cell Signaling Technology	Cat# 4947
NF-κB p65 Rabbit mAb	Cell Signaling Technology	Cat# 8242

Phospho-NF-κB p65 (Ser536) Rabbit mAb	Cell Signaling Technology	Cat# 3033
TBK1/NAK Rabbit mAb	Cell Signaling Technology	Cat# 38066
Phospho-TBK1/NAK (Ser172) Rabbit mAb	Cell Signaling Technology	Cat# 5483
Anti-TLR3 antibody	Abcam	Cat# ab13915
Anti-PKR antibody	Abcam	Cat# ab184257
Anti-MAVS antibody	Abcam	Cat# ab189109

Table S3. Antibodies used for FACS, IF and IHC analysis

**ANTIBODY**	**SOURCE**	**IDENTIFIER**
PE/Cyanine7 anti-mouse CD11c antibody	Biolegend	Cat# 117317
PE/Cyanine5 anti-mouse CD40 antibody	Biolegend	Cat# 124617
Brilliant Violet 510™ anti-mouse CD86 antibody	Biolegend	Cat# 105039
PE anti-mouse CD80 antibody	Biolegend	Cat# 104707
FITC anti-mouse H-2Kb antibody	Biolegend	Cat# 116505
PE/Cyanine5 anti-mouse I-A/I-E antibody	Biolegend	Cat# 107611
PE anti-mouse H-2Kb S IINFEKL antibody	Biolegend	Cat# 141603
PE/Cyanine7 anti-mouse CD45 antibody	Biolegend	Cat# 157205
Brilliant Violet 421™ anti-mouse CD45 antibody	Biolegend	Cat# 103133
PerCP/Cyanine5.5 anti-mouse CD3 antibody	Biolegend	Cat# 100217
FITC anti-mouse CD8a antibody	Biolegend	Cat# 100705
PE anti-mouse CD4 antibody	Biolegend	Cat# 100407
Brilliant Violet 421™ anti-human/mouse Granzyme B Recombinant antibody	Biolegend	Cat# 396414
Brilliant Violet 510™ anti-mouse IFN-γ antibody	Biolegend	Cat# 505841
PE anti-mouse/human CD11b antibody	Biolegend	Cat# 101207
FITC anti-mouse F4/80 antibody	Biolegend	Cat# 123107

Brilliant Violet 421™ anti-mouse CD206 (MMR) antibody	Biolegend	Cat# 141717
FITC anti-mouse CD25 antibody	Biolegend	Cat# 101907
Brilliant Violet 421™ anti-mouse FOXP3 antibody	Biolegend	Cat# 126419
Brilliant Violet 421™ anti-mouse Ly-6C antibody	Biolegend	Cat# 128031
Brilliant Violet 570™ anti-mouse Ly-6G antibody	Biolegend	Cat# 127629
Brilliant Violet 421™ anti-mouse CD69 antibody	Biolegend	Cat# 104527
Brilliant Violet 421™ anti-mouse CD206 (MMR) antibody	Biolegend	Cat# 141717
Calreticulin Rabbit mAb (AF 488 Conjugate)	Cell Signaling Technology	Cat# 62304S
Rabbit mAb IgG Isotype control (AF 488 Conjugate)	Cell Signaling Technology	Cat# 2975S
FITC anti-mouse CD103 antibody	Biolegend	Cat# 121419
CD8α Rabbit mAb (Mouse Specific)	Cell Signaling Technology	Cat# 60168
Cleaved Caspase-3 (Asp175) Rabbit mAb	Cell Signaling Technology	Cat# 9664
Anti-CD103 antibody	Abcam	Cat# ab224202
Anti-DNA mAb	Progen	Cat# 61014
cGAS Rabbit mAb	Biorbyt	Cat# rb1149894
Anti-COX IV-AF647 antibody	ImmunoWay	Cat# YM2009
Goat anti-Mouse IgG, IgM (H+L) (AF 488)	Invitrogen	Cat# A-10680
Goat Anti-Rabbit IgG H&L (Alexa Fluor® 405)	Abcam	Cat# ab175652

Table S4. Primers for RT-qPCR

qPCR: Mouse IFNB1 forward	ATGGAGATGACGGAGAAGATGC
qPCR: Mouse IFNB1 reverse	TTCAGAAACACTGTCTGCTGGT
qPCR: Mouse TNF forward	CCTCTCATCAGTTCTATGGCCC
qPCR: Mouse TNF reverse	GTCTTTGAGATCCATGCCGTTG

qPCR: Mouse ISG15 forward	GTCTTACCCTTTCCAGTCTGGG
qPCR: Mouse ISG15 reverse	TACAGTCTGCGTCAGAAAGACC
qPCR: Mouse CXCL10 forward	TCATTTTCTGCCTCATCCTGCT
qPCR: Mouse CXCL10 reverse	TCTGCAAGCTGAAGGGATTTCT
qPCR: Mouse IFIT1 forward	ATGTCAGAAGGAAGAGTGCAGG
qPCR: Mouse IFIT1 reverse	TGCATGCTACCTGAGTTGACAT
qPCR: Mouse CCL5 forward	TTTCTTCCTTCTCCCAGATGGC
qPCR: Mouse CCL5 reverse	TATACCCCACAGAGGAACCCAT
qPCR: Mouse IRF9 forward	GGAAAAGCACAAAGATGGGGAC
qPCR: Mouse IRF9 reverse	GCTTGCATGGTGATTTCTGGTT
qPCR: Mouse GAPDH forward	TGGCCTCCAAGGAGTAAGAAAC
qPCR: Mouse GAPDH reverse	ATTCAAGAGAGTAGGGAGGGCT

Table S5. sgRNA sequence for knocking out the indicated proteins.

sgRNA: Mouse cGAS forward	CGAGGCGCGGAAAGTCGTAA
sgRNA: Mouse cGAS reverse	TTACGACTTTCCGCGCCTCG
sgRNA: Mouse STING forward	GTACCCAATGTAGTATGACC
sgRNA: Mouse STING reverse	GGTCATACTACATTGGGTAC
sgRNA: Mouse MAVS forward	GCCACCAGACATCCTCGCGA
sgRNA: Mouse MAVS reverse	TCGCGAGGATGTCTGGTGGC
sgRNA: Mouse TLR3 forward	GTACTGCTCATTCACATCGA
sgRNA: Mouse TLR3 reverse	TCGATGTGAATGAGCAGTAC
sgRNA: Mouse PKR forward	CGTTCGTCTAAAAGGCAGAG
sgRNA: Mouse PKR reverse	CTCTGCCTTTTAGACGAACG
sgRNA: Mouse TDP-43 forward	ACTGGTCACTCGAAAGGGTT

sgRNA: Mouse TDP-43 reverse	AACCCTTTCGAGTGACCAGT

Table S6. siRNA sequence for knocking down the indicated proteins.

siRNA: Mouse Tln2 forward	CTGTGAAGGCAGTGGAAGATT
siRNA: Mouse Tln2 reverse	TCTTCCACTGCCTTCACAGTT
siRNA: Mouse Myo18a forward	CTGTGAAGGCAGTGGAAGATT
siRNA: Mouse Myo18a reverse	TCTTCCACTGCCTTCACAGTT
siRNA: Mouse Myh10 forward	CTGTGAAGGCAGTGGAAGATT
siRNA: Mouse Myh10 reverse	TCTTCCACTGCCTTCACAGTT
siRNA: Mouse Tdp43 forward	CTGTGAAGGCAGTGGAAGATT
siRNA: Mouse Tdp43 reverse	TCTTCCACTGCCTTCACAGTT
siRNA: Mouse Tmem43 forward	CTGTGAAGGCAGTGGAAGATT
siRNA: Mouse Tmem43 reverse	TCTTCCACTGCCTTCACAGTT
siRNA: Mouse Mtif2 forward	CTGTGAAGGCAGTGGAAGATT
siRNA: Mouse Mtif2 reverse	TCTTCCACTGCCTTCACAGTT
siRNA: Mouse Ssr1 forward	CTGTGAAGGCAGTGGAAGATT
siRNA: Mouse Ssr1 reverse	TCTTCCACTGCCTTCACAGTT
siRNA: Mouse Ndufa7 forward	CTGTGAAGGCAGTGGAAGATT
siRNA: Mouse Ndufa7 reverse	TCTTCCACTGCCTTCACAGTT
siRNA: Mouse Dad1 forward	CTGTGAAGGCAGTGGAAGATT
siRNA: Mouse Dad1 reverse	TCTTCCACTGCCTTCACAGTT
siRNA: Mouse Mif forward	CTGTGAAGGCAGTGGAAGATT
siRNA: Mouse Mif reverse	TCTTCCACTGCCTTCACAGTT
